# Horizontal transfer of bacterial polyphosphate kinases to eukaryotes: implications for the ice age and land colonisation

**DOI:** 10.1186/1756-0500-6-221

**Published:** 2013-06-05

**Authors:** Michael P Whitehead, Paul Hooley, Michael R W Brown

**Affiliations:** 1 School of Applied Sciences, University of Wolverhampton, Wolverhampton, UK; 2 UCL School of Pharmacy, London WC1N 1AX, UK

**Keywords:** Polyphosphates, Horizontal gene transfer, PPK1, PPK2, Stress responses

## Abstract

**Background:**

Studies of online database(s) showed that convincing examples of eukaryote PPKs derived from bacteria type PPK1 and PPK2 enzymes are rare and currently confined to a few simple eukaryotes. These enzymes probably represent several separate horizontal transfer events. Retention of such sequences may be an advantage for tolerance to stresses such as desiccation or nutrient depletion for simple eukaryotes that lack more sophisticated adaptations available to multicellular organisms. We propose that the acquisition of encoding sequences for these enzymes by horizontal transfer enhanced the ability of early plants to colonise the land. The improved ability to sequester and release inorganic phosphate for carbon fixation by photosynthetic algae in the ocean may have accelerated or even triggered global glaciation events. There is some evidence for DNA sequences encoding PPKs in a wider range of eukaryotes, notably some invertebrates, though it is unclear that these represent functional genes.

Polyphosphate (poly P) is found in all cells, carrying out a wide range of essential roles. Studied mainly in prokaryotes, the enzymes responsible for synthesis of poly P in eukaryotes (polyphosphate kinases PPKs) are not well understood. The best characterised enzyme from bacteria known to catalyse the formation of high molecular weight polyphosphate from ATP is PPK1 which shows some structural similarity to phospholipase D. A second bacterial PPK (PPK2) resembles thymidylate kinase. Recent reports have suggested a widespread distribution of these bacteria type enzymes in eukaryotes.

**Results:**

On – line databases show evidence for the presence of genes encoding PPK1 in only a limited number of eukaryotes. These include the photosynthetic eukaryotes *Ostreococcus tauri*, *O. lucimarinus*, *Porphyra yezoensis, Cyanidioschyzon merolae* and the moss *Physcomitrella patens,* as well as the amoeboid symbiont *Capsaspora owczarzaki* and the non-photosynthetic eukaryotes *Dictyostelium* (3 species), *Polysphondylium pallidum* and *Thecamonas trahens*. A second bacterial PPK (PPK2) is found in just two eukaryotes (*O. tauri* and the sea anemone *Nematostella vectensis*). There is some evidence for PPK1 and PPK2 encoding sequences in other eukaryotes but some of these may be artefacts of bacterial contamination of gene libraries.

**Conclusions:**

Evidence for the possible origins of these eukaryote PPK1s and PPK2s and potential prokaryote donors via horizontal gene transfer is presented. The selective advantage of acquiring and maintaining a prokaryote PPK in a eukaryote is proposed to enhance stress tolerance in a changing environment related to the capture and metabolism of inorganic phosphate compounds. Bacterial PPKs may also have enhanced the abilities of marine phytoplankton to sequester phosphate, hence accelerating global carbon fixation.

## Background

Recent reviews have proposed a widespread occurrence of horizontally transferred bacterial type polyphosphate kinase enzymes in eukaryotes [1]. Inorganic polyphosphate (poly P) has been present since pre-biotic times [2] and has been proposed as an energy distributor in a pre-ATP world [3]. Poly P is found in organisms that represent species from each domain in nature: Eukarya, Archaea and Bacteria [4-6]. Studied mainly but not exclusively in prokaryotes, poly P and its associated enzymes are vital in diverse basic metabolism, in at least some structural functions and, notably, in stress responses [7]. These numerous and unrelated roles for poly P are probably the consequence of its presence in life-forms from early in evolution [8]. The genomes of many bacterial species, including human pathogens, encode a homologue of a major poly P synthetic enzyme, polyphosphate kinase 1 (PPK1) based on a phospholipase D structure [9]. Loss of PPK1 produces reduced poly P levels, and deletion of the *ppk1* gene in pathogens also results in a loss of virulence towards protozoa and animals [7]. A second PPK activity in bacteria, PPK2, is related to thymidylate kinase [10,11]. PPK2 is distinguished from PPK1 by its preference for utilising poly P in the reversible generation of GTP. Polyphosphate-AMP-phosphotransferase (PAP) uses poly P to phosphorylate AMP to ADP [8]. Other enzymes that influence accumulation of poly P are the hydrolytic enzymes: exopolyphosphatase (PPX) that releases Pi from the ends of poly P [12] and endopolyPases (PPN) that cleave poly P to progressively shorter chains. These enzymes together maintain poly P metabolism and catabolism in bacteria. Poly P metabolism is less well characterised in eukaryotic systems. In *Dictyostelium discoideum* a PPK activity (DdPPK2) based on three actin like proteins has been documented [13]. Hothorn *et al.* 2009 [14] identified a PPK activity associated with a fourth class of enzyme, a vacuolar transport chaperone (VTC), which has a distribution largely limited to simple eukaryotes. Intriguingly, Reusch *et al.* (1997) [15] assigned a PPK activity to a Ca^2+^ ATPase activity in humans. Recently Lonetti *et al.* (2011) [16] demonstrated *Saccharomyces cerevisiae* to have undetectable levels of poly P in mutants unable to produce inositol pyrophosphates. Hence, a range of families of enzymes have been shown to have PPK activity. The enzymes responsible for poly P synthesis in most eukaryotes remain unidentified with the exceptions of an actin related protein in *Dictyostelium discoideum*[13], vacuolar transport chaperones in *Saccharomyces cerevisiae*[14] and a Ca^2+^ ATPase in *Homo sapiens*[15]. In this respect, the hypothesis that bacteria – like PPK enzymes based on phospholipase D [9] or thymidylate kinase [10,11] exist in eukaryotes, requires investigation.

Horizontal gene transfer (HGT) is now recognised as an important force in eukaryote genome evolution [17]. Hooley *et al.* (2008) [18] summarised evidence that suggested a very limited distribution of bacterial type PPK1 and PPK2 enzymes in a small number of eukaryotes. Rao *et al.* (2009) [1] have claimed evidence for a surprisingly wide potential distribution of the bacteria type PPK1 and PPK2 in eukaryotes. Computer aided bioinformatics techniques can exploit genome project databases swiftly to summarise likely candidates for PPK activity. Similarity search tools such as BLAST [19] and multiple alignment programs like Clustal W/X, TCoffee and MUSCLE [20-22] allow rapid comparisons of sequence data. Phylogenetic analyses [23,24] can infer evolutionary relationships between DNA or protein sequences. The current paper aims to examine the evidence for bacteria type polyphosphate kinases in eukaryotes and to consider their relationships to possible donor prokaryotes. The possible selective advantages in acquiring such prokaryote genes are discussed.

## Results

### Bacteria type PPKs on interpro

An initial analysis of PPK’s listed on Interpro was carried out to eliminate any sequences with weak support for their annotations. Table 1 reports annotations for PPK1 and PPK2 for eukaryotes held under 4 different Interpro accession numbers. The annotations of three *Populus trichocarpa* (B9PBE1, BPDP9 and B9NJ30) accessions are questionable. The encoding sequence for B9PBE1 (PPK1) has previously been reported to show 100% identity to DNA from bacteria [18]. Similarly, BLASTn searches at NCBI reveal 98% and 100% identity over the entire coding regions of BPDP9 and B9NJ30 (both PPK2) respectively to *Delftia* and *Cupriavidus* bacteria. A PPK1 (F4PF87) is listed on Interpro for the chytrid *Batrachochytrium dendrobatidis* strain JAM81 (http://genome.jgi-psf.org/Batde5/Batde5.home.html). This accession lacks introns and is absent from a second strain JEL423 (http://www.broadinstitute.org/annotation/genome/batrachochytrium_dendrobatidis/MultiHome.html). It is annotated only as a predicted protein on a short scaffold without any determined EST’s. However, it shows only limited DNA similarity to bacterial genomes (best match 68% of 1728 bases identical to *Bacillus cytotoxicus*), which may explain why the sequence was not annotated out of the current draft of the genome sequence. These doubtful annotations were excluded from further analysis.

**Table 1:** A summary of bacteria type polyphosphate kinase accessions for eukaryotes on Interpro (12/2/12)

**IPR003414 (PPK1)**	
A2VBB6	*Porphyra yezoensis*
A4RQI1	*Ostreococcus lucimarinus*
A9U2N0	*Physcomitrella patens*
B9PBE1	*Populus trichocarpa*
Q01H21	*Ostreococcus tauri*
Q2MEV6	*Physcomitrella patens*
D3B5H9	*Polysphondylium pallidum*
Q54BM7	*Dictyostelium discoideum*
E9CFK0	*Capsaspora owczarzaki*
F4PF87	*Batrachochytrium dendrobatidis*
**IPR022488 (PPK2 related)**	
A8DVD6	*Nematostella vectensis*
B9NJ30	*Populus trichocarpa*
B9PDP9	*Populus trichocarpa*
Q015Y3	*Ostreococcus tauri*
**IPR022486 (PPK2, PAO141)**	
Q015Y3	*Ostreococcus tauri*
**IPR016898 (PPK2)**	
Q015Y3	*Ostreococcus tauri*
A8DVD6	*Nematostella vectensis*

Extensive searches of a number of other databases revealed only a small number of other PPK1 enzymes and no further PPK2 matches. Even using a high automatic BLASTp search threshold of e < 10 or 1, only compelling matches with tiny (1e^-15^ or less) e-values were generated for visual confirmation. On NCBI, 73 fungi, 40 protozoa, 27 insects and 6 nematodes gave no additional hits with the exception of a *Dictyostelium fasciculatum* PPK1 (Genbank: EGG21828.1). No additional PPKs were found in 25 species of Viridoplantae, two species of diatoms (*Thalassiosira pseudonana* and *Phaeodactylium tricornutum*), the haptophyte *Emiliana huxleyi*, or the lycophyte *Selaginella huxleyi*. Additional and convincing matches to PPK1 were found in the amoeboid symbiont *Capsaspora owczarzaki* and the protistan *Thecamonas trahens*. The red algal species *Cyanidioschyzon merolae* and *Porphyra yezoensis* both show convincing evidence of PPK1 enzymes of bacterial origin. The *P. yezoensis* PPK1 sequence is 913 amino acids in length but is incomplete at the N terminal as it is based on an incomplete mRNA sequence.

Additional file 1 shows complete alignments of each of the eukaryote PPK1 enzymes compared with prokaryote and archaea controls. Within this, it can be seen that the *C. owczarzaki* protein shows distinct, unique inserts causing non-alignment. Such large and numerous inserts may have a disproportionate affect on phylogenetic analysis; hence this sequence was excluded from further analysis. Eukaryote PPK1 enzymes are characterised by extensive N terminals making them longer than the bacterial counterparts [18]. In addition a PPK1 was identified in the annotated but incomplete genome of *Ostreococcus* sp.RCC 809 (not included in analysis - http://www.jgi.doe.gov).

**Figure 1:**
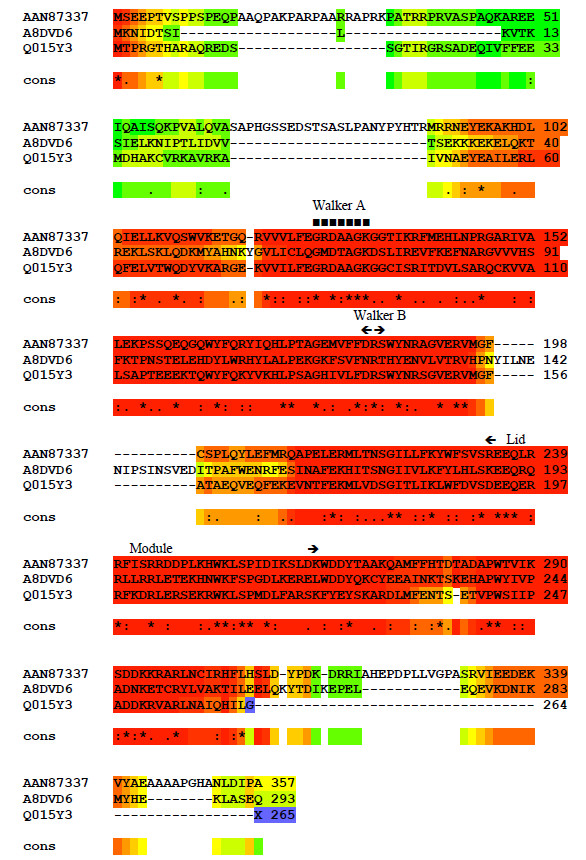
**TCoffee analysis of bacteria type PPK2 enzymes.***Pseudomonas aeruginosa* accession (GenBank AAN87337) compared to Interpro: A8DVD6 (*Nematostella vectensis*) and Interpro: Q015Y3 (*Ostreococcus tauri*).Square symbols ■ indicate putative P loop (ATP/GTP binding site motif [10])/Walker A motif with Walker B and lid modules indicated [11]. Other residues (*) perfectly conserved (cons), (:) very similar, (.) similar. Warmer colours (red and orange) show best – matching regions through to cooler colours (green, blue) with poorer alignment.

Table 2 summarises the intron density of these eukaryote *ppk* genes. The recently completed *Dictyostelium purpureum* genome (http://genome.jgi-psf.org/Dicpu1/Dicpu1.home.html) was searched using BLASTp to reveal an ortholog of the *D. discoideum* PPK1 protein (Dicpu1_45674) with a single intron [25]. Two *ppk1* type genes are annotated in *Physcomitrella patens*. They show around 84% nucleotide identity with each other, presumably reflecting a common origin and/or duplication of a single sequence. These two genes encode proteins that differ in length by 71 amino acids with the key differences appearing in the extended N terminal region [18]. Remarkably 21 and 22 introns are annotated in these two *ppk1* genes (Table 3; http://genome.jgi-psf.org/Phypa1_1/Phypa1_1.home.html). *Capsaspora owczarzaki ppk1* (CAOG_06840T0) and *Thecamonas trahens ppk1* (AMSG_11662.2) both show three introns. No introns are annotated in the *ppk* genes from the two *Ostreococcus* species (http://genome.jgi-psf.org/Ostta4/Ostta4.home.html; http://genome.jgi-psf.org/Ost9901_3/Ost9901_3.info.html) or the *C. merolae ppk1* gene. Figure 1 illustrates the identities between the two eukaryote PPK2 enzymes and a model prokaryote. Extensive conservation is revealed throughout the bulk of the sequences with some increased variability observed particularly at the N terminus.

**Table 2:** **Summary of intron density in eukaryote *ppk1 *and *ppk2 *genes (number per gene)**

***Gene***	**Species**
***ppk1***	
21, 22	*Physcomitrella patens*
0	*Ostreococcus lucimarinus*
0	*O. tauri*
0	*Dictyostelium discoideum*
1	*D. purpureum*
4	*Polysphondylium pallidum*
3	*Capsaspora owczarzaki*
0	*Batrachochytrium dendrobatidis*
3	*Thecamonas trahens*
***ppk2***	
0	*Nematostella vectensis*
0	*O. tauri*

**Table 3:** **Characteristics of introns of *Physcomitrella patens *for genes encoding PPK1s (Interpro accession codes) compared to means for genome**[45]

**PPK1**	**Introns**	**Mean intron length (bp +/− S.E.)**	**Total intron length per gene (bp)**	**Total exon length per gene (bp)**
Q2MEV6 (973 amino acids)	21	151 +/− 10	3160	29
A9UZN0 (902 amino acids)	22	168 +/− 10	3688	2709
*P. patens* mean	5.7 +/− 0.5	262 +/− 12	1330 +/− 113	1448 +/− 78

### Phylogenies of eukaryote bacteria type PPK1 and PPK2

**Figure 2:**
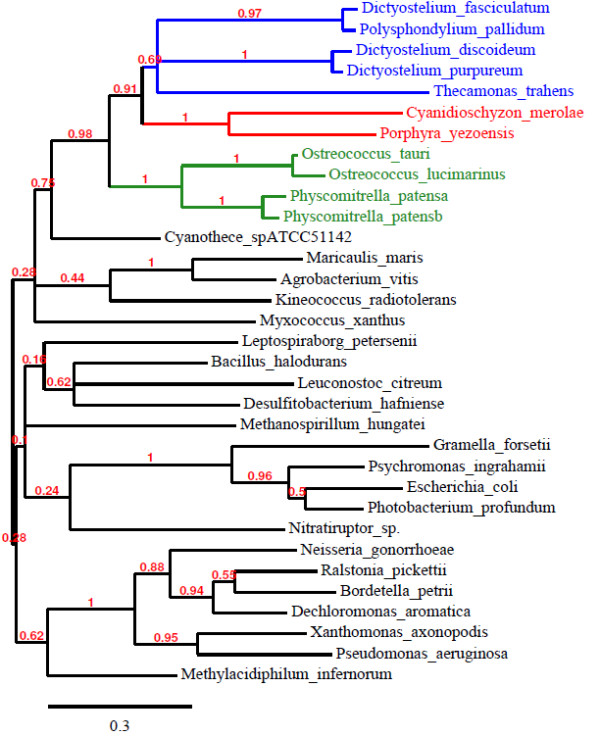
**Phylogenetic analysis of PPK1.** Numbers on the branches indicate bootstrapping values out of 100 calculated for maximum likelihood (expressed as a fraction of 1). Those branches showing less than 10% have been collapsed. Colours of eukaryotes indicate major taxonomic groupings (blue- non-photosynthetic eukaryotes, green- plants and green algae, red- red algae).

**Figure 3:**
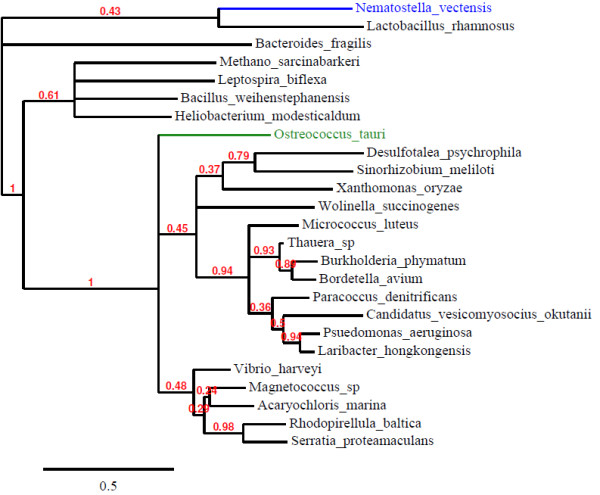
**Phylogenetic analysis of PPK2.** Numbers on the branches indicate bootstrapping values out of 100 calculated for maximum likelihood (expressed as a fraction of 1). Those branches showing less than 10% have been collapsed. Colours of eukaryotes indicate major taxonomic groupings (blue-metazoa, green- plants and green algae).

The TreeDyn analysis of PPK1 is shown in Figure 2. This shows that the eukaryotic sequences group together consistent with their taxonomic groupings. The bootstrapping numbers indicate that these groupings can be relied upon with a high degree of confidence. The most closely related bacterial species to all the eukaryotic PPK1 sequences consistently came from the cyanobacteria group (Figure 2). When additional top matching (e-value = 0 from BLASTp searches) cyanobacteria are included in the analysis this strengthens the association of this group of bacteria to the eukaryotic PPKs (Additional file 2). When additional bottom matching cyanobacteria (but still with very low e-values of approximately 1e^-140^) are included in the analysis no such association is seen (Additional file 3), indicating that the eukaryotic PPK1s share an origin with a subset of the cyanobacteria. When the analysis is repeated without the eukaryotic specific N extension the support for the association with *Cyanothece* is increased. Figure 3 shows the TreeDyn view of PPK2, which indicates three distinct groupings which are not necessarily associated with the taxonomic classifications. The results show that the eukaryotes consistently cluster with separate groups of bacterial PPK2s.

### Invertebrates

Blastp searches for *E. coli* PPK1 and *P. aeruginosa* PPK2 produced no significant similarities against 28 completed arthropod genomes. tBlastn searches gave four matches to each sequence which are summarised in Table 4. The accessions for each of the *Aedes aegypti* matches described the sequences as of “probable bacterial origin” and none of them matched verified transcripts. DNA sequences for the remaining four sequences were searched against the entire NCBI database via Blastn. In each case, excellent matches to bacterial DNA sequences were found with no other matching arthropod DNA. ABLF02002165.1 gave 79% identity over 1405 bases (e = 0) to a *ppk1* from *Staphylococcus saprophyticus*. EST/transcript searches on AphidBase revealed only two short matches (28 out of 32 bases at e = 0.055). ACJG01018676.1 gave 69% identity over 644 bases (e = 2 e^-57^) to a *ppk1* from *Conexibacter woesei.* In this case, EST data on the *Daphnia* JGI site supported a single, intronless 807 bp long gene (e = 0). ABJB010687643.1 gave 97% identity over 1164 bases (e = 0) to a *Pseudomonas fluorescens* polyphosphate AMP phosphotransferase gene (*ppk2*). Only short EST/transcript matches to just 23 bases (e value 0.51) could be found on the *Ixodes* database for this sequence. Finally, ABJB010847895.1 gave 71% identity over 736 bases (e = 1 e^-92^) to a short chain dehydrogenase/reductase from *Burkholderia phymatum* and one perfect transcript match to all 762 bases (e = 0) on the *Ixodes* database. This sequence matched to an intronless gene (ISCW024221) described as a reductase.

**Table 4:** **Summary of *E. coli *PPK1 and *P. aeruginosa *PPK2 tBlastn searches against NCBI Arthropoda database (28 species) displaying hits with e-values < 0.05**

**Species**	**Accession**	**E value**
**PPK1**		
*Aedes aegypti* strain Liverpool cont 1.27756	AAGE02027756.1	2 e^-176^
*Aedes aegypti* strain Liverpool cont 1.32234	AAGE02032234.1	1 e^-106^
*Acyrthosiphon pisum* strain LSR1 cont 2200	ABLF02002165.1	3 e^-90^
*Daphnia pulex* DAPPU scaffold_13832_cont 18676	ACJG01018676.1	1 e^-14^
**PPK2**		
*Aedes aegypti* strain Liverpool cont 1.29024	AAGE02029024.1	3 e^-83^
*Aedes aegypti* strain Liverpool cont 1.30944	AAGE02030944.1	3 e^-38^
*Ixodes scapularis* strain Wikel colony g cont_1108378508994	ABJB010687643.1	5 e^-22^
*Ixodes scapularis* strain Wikel colony g cont_1108379235394	ABJB010847895.1	0.083

## Discussion

Nearly 200 eukaryote genomes have been examined in the present work for evidence of bacteria type PPK1 and PPK2. No single database contains a definitive list of eukaryote bacteria type PPKs but we can conclude that relatively few eukaryotes possess these enzymes (Table 1, Additional file 1). Hooley *et al.* (2008) [18] reported extensive conservation of structure between bacterial PPK1s and their eukaryote counterparts. Here we demonstrate a similar degree of conservation between the two eukaryote examples of PPK2 and bacterial counterparts (Figure 1). There is therefore a taxonomically discontinuous distribution of a limited number of bacterial type PPKs in diverse simple eukaryotes. The most parsimonious explanation for such eukaryote *ppk* genes, several of which contain no introns suggesting prokaryote origins, is a number of independent horizontal transfer events from bacteria.

At the outset, it was important to eliminate possible artefacts from the analysis. There are several clear examples of likely incorrect identifications of PPK encoding genes. For example the 100% DNA sequence identity of the proposed *Populus ppk1* and *ppk2* with *Ralstonia/Delftia* seems an obvious case of bacterial contamination*.* Several hits to bacteria – type PPK1s have been claimed for insects [1,26]. These exciting suggestions must be examined in the light of the possible contamination of gene banks with bacterial sequences [27]. Conversely it is possible that vector search programs may erroneously eliminate real examples of horizontal transfers. It is important to consider that DNA sequencing and annotation errors may give misleading gene descriptions [28]. Table 4 summarises a relatively small number of arthropod matches that identify potential PPK1 and PPK2 encoding sequences. Of these, four have clearly been identified as likely bacterial contaminants by the original workers. Just two of the remaining four have some EST support for their presence as genuine eukaryote genes. Their lack of similarity to any other arthropod DNA sequences and high DNA identity to bacteria still makes their annotation questionable.

Host/parasite relationships provide obvious opportunities for gene exchange [17]. However, in a genome project there is the potential for mistaking a horizontally transferred gene for a bacterial DNA contaminant acquired during gene bank construction [29]. The *Wolbachia* insect symbiont has integrated 30% of its genome into the *Callosobruchus* beetle genome; most of these genes are disrupted and transcriptionally inactive [30]. Klasson *et al.* 2009 [31] demonstrated the expression of *Wolbachia* genes in *Aedes* mosquito. These observations may be consistent with early Blastn reports of *ppk1* matches at the DNA level to some invertebrates [1,26]. However, bacterial DNA may be horizontally transferred but not active as a PPK1 product. The twenty *Wolbachia* genomes available on NCBI were searched via BLASTp using the *E. coli* PPK1 (NP_416996 ) and *P. aeruginosa* PPK2 (NP_248831) protein sequences. No significant PPK1 (lowest e – value 2.3, just 29% identity over 34 amino acids) or PPK2 (two hits at e – value < 0.05, best being e = 0.042 with just 27% identity over 82 amino acids) matches were found. The source of potential PPK encoding sequences, whether active or not, in invertebrates remains a puzzle. Claims for the widespread occurrence of bacteria type PPKs in eukaryotes [1] are overstated and these enzymes show a much more restricted distribution.

Figure 2 demonstrates quite distinct clusters of PPK1 enzymes in eukaryotes. These clusters are consistent with the taxonomic groupings of these eukaryotes. *P. patens*, *O. tauri* and *O. lucimarinus* form one group (green plants and green algae), the three *Dictyostelium* species, *P. pallidum* and *T. trahens* (non-photosynthetic eukaryotes) a second group, with *P. yezoensis* and *C. merolae* (red algae) forming a third group. All of these groups are well supported by bootstrapping values. The most obvious donor for horizontally transferred PPK1s in eukaryotes is an ancestor in common with the cyanobacteria as shown by the association of the *Cyanothece* sp. PPK1 with the eukaryotic grouping. The cyanobacteria formed the original endosymbionts generating chloroplasts. The two *Ostreococcus* species are generally considered to be very divergent, with an average of only 70% amino acid identity between orthologous proteins, making *O. tauri* and *O. lucimarinus* amongst the most dissimilar members of the same genus in any eukaryote [32]. The most parsimonious explanation may be the acquisition of PPK1 when *Ostreococcus* and *Physcomitrella* last shared a common ancestor, with subsequent losses in other lines. Attempting to trace a common or single origin of a specific *ppk* gene may be unrealistic, particularly in light of the complex evolution of algae with the potential for secondary horizontal gene transfer events [33].

There are only two convincing eukaryote PPK2s found on the Interpro database (Figure 1). Phylogenetic analysis suggests that these two have quite different origins (Figure 3). Interestingly, *O. lucimarinus* appears not to have a *ppk2* – presumably the *O. tauri* example was gained after the species separated or a *ppk2* sequence inherited from a common ancestor was subsequently lost by one species. Derelle *et al.* 2008 [34] describe one possible candidate virus, OtV5, as an agent of horizontal transfer in this genus. Raymond and Blankenship (2003) [35] emphasise the importance of HGT in evolution of eukaryotic algae with endosymbiosis extending beyond the original event of engulfment of cyanobacteria to create plastids to include acquisition of genes from other algae at other times. Rohwer and Thurber (2009) [36] give further examples of HGT into metazoans within the marine environment including viral vectors moving genes between animals and plants.

It is also important to highlight those groups of organisms which are notable by their absence from the short list of eukaryote PPKs. Horizontally transferred genes have been shown to affect the metabolism of numerous fungal species [37]. Yet no examples of PPK1 or PPK2 were observed in the annotated or partially completed 116 genomes of fungi investigated here. However, since fungi do not engulf organisms via phagotrophy, it may suggest an additional clue to the origin of the horizontally transferred PPKs in other eukaryotes. Similarly, most simple eukaryotes and almost all photosynthetic organisms examined did not contain these bacteria type enzymes.

Horizontally transferred genes from eubacteria would be expected at least initially to contain no introns. Assuming that the bacteria type PPK1 and PPK2 enzymes have been acquired from bacteria, how and why do horizontally transferred genes acquire introns? Table 2 reveals that some eukaryote *ppk1* and *ppk2* examples are indeed intron free. Spliceosomal introns are typically found in nuclear genomes, and their presence indicates a major role in evolution, although no overall general function is known. It is known that organisms with shorter life cycles tend to have less intronization perhaps reflecting selection for reduced processing time for mRNAs. Intron length has been positively correlated to gene expression in unicellular eukaryotes but negatively correlated in multicellular eukaryotes whilst mildly deleterious elements may accumulate in more complex organisms that have small populations [38,39]. In the examples shown in the present work there is a range from *ppk1* genes with no introns (such as in *Ostreococcus* and *D. discoideum*) to the 21 and 22 introns found in *P. patens* (Table 2). Neither example of eukaryote *ppk2* has introns (*O. tauri, N. vectensis*). Rotifers, which also predate bacteria, have acquired many bacterial genes and whilst some are defective, others are expressed and these may include genes with introns [40]. This compares with relatively little evidence of intron gain in *Entamoeba* horizontally transferred genes [41] or in the recent evolutionary past of higher plants [42]. There is no single source of data expressing intron density for each of these eukaryote species in a common format, with some authors and databases quoting introns per gene, introns per transcript, introns per kb of coding sequence or introns per spliced gene. The *C. merolae* and *D. discoideum* genomes have means of just 0.005 and 1.31 introns per gene respectively [39] so absence or a low number of introns in their *ppk* genes is not remarkable. A possible mechanism of intron gain could be increased transposon activity that activated double stranded DNA repair [43].

In *P. patens* an accepted horizontally transferred glycerol/water channel gene has 5 introns [44]. There are four recognised domains for PPK1 [9] so multiple introns are unlikely to have evolved as a means to promote domain shuffling. Stenoien (2007) [45] suggests that highly expressed genes in *P. patens* have shorter introns than genes with low expression levels and so acquisition of small introns may reflect mechanisms to regulate gene expression (Table 3). It has been suggested that there has been an ancestral duplication of the *P. patens* genome [27,46] with essential genes such as *rad51* (recombination repair) being present in two copies, thus allowing pseudoallelism and the protection of an essential function in the gametophyte (haploid) plant. Hence, the presence of two copies of a *ppk1* gene in this species suggests an essential function. Table 3 implies that the two *P. patens ppk1* genes have an unusually high number of introns. However, Csuros *et al.* 2011 [43] suggest that this species has a mean of 5.5 introns per kb of coding sequence. On this basis, the apparently large number of introns may, at least partially, reflect the unusually large size of these two genes for the species. Sucgang *et al.* 2011 [47] describe mean intron values of 1.9 and 1.5 per spliced gene for *D. discoideum* and *D. purpureum* respectively. *D. discoideum* and *D. purpureum* diverged approximately 400 million years ago [47] so the acquisition of PPK1 in these and the other slime moulds presumably predates this speciation event. The colonisation of the land by plants around 470 million years ago was followed by the divergence of the line leading to *Physcomitrella* from that leading to *Selaginella* and higher plants about 430 million years ago [48]. Hence, it is reasonable to suggest that one horizontal transfer event may have occurred in a common ancestor of these species and the genes have been maintained by common selective pressures. In individual species such as *Selaginella* the *ppk* genes have subsequently been lost. Although this is the most parsimonious explanation, the occurrence of multiple acquisitions and losses should not be ruled out.

What are the advantages to a eukaryote in maintaining a bacteria-type PPK? As poly P is found in all cells there must be alternative mechanisms for manufacture in eukaryotes, perhaps based on the actin related PPK3 system [18]. Horizontally acquired PPK1 or PPK2 must replace or supplement such native enzymes. Indeed, the primitive red alga *C. merolae* has a VTC1p homologue in a poly P containing vacuole. Poly P is a key store of phosphate in this acid and heat tolerant species rather than phytic acid commonly found in higher plants [49]. Similarly poly P is the stored form of phosphate in green algae [50], hence acquiring PPK1 or PPK2 may provide a greater flexibility in nutrient stress responses in algal cells. *O. tauri* is a unicellular green alga that is an important member of the global phytoplankton and has cell dimensions of around 1 μm diameter, equivalent to a prokaryotic cell. In *O. tauri* nitrogen starvation results in an increase in PPK activity [51]. So poly P accumulation via enhanced PPK activity is clearly a valuable response to nutrient depletion stress, possibly to maintain phosphate reserves. Such activity may withdraw more reactive and soluble phosphate molecules from metabolism or potential efflux from a tiny cell, like *O. tauri*or *C. merolae*, with a high ratio of surface area to volume.

Simple multicellular eukaryotes may face periods of water immersion followed by desiccation. For example, mosses such as *P. patens* and slime moulds both have a need to escape aqueous environments to sporulate [52]. Desiccation tolerance in individual cells and tissues is required in plants such as *P. yezoensis* and *P. patens*, which lack the efficient vascular systems of higher plants [27]. Under such circumstances there may be selection pressure to use horizontally transferred genes available in the surrounding bacterial population. Higher plants have more sophisticated water transport, compatible solute manufacture and waxy cuticles so that individual cells are no longer desiccated. Similarly, filamentous fungi have thick chitinous walls and the VTC PPK system [14], so may be more desiccation tolerant anyway. Hence slime moulds and mosses may represent special cases of incomplete adaptations to dry land colonisation.

Eukaryote PPK1s are characterised by being of higher mass than prokaryote homologs [18] – N terminal extensions perhaps reflect differences in targeting the enzyme, e.g. to a membrane or subcellular compartment, or in assuming quaternary structures. However, Target P and Signal P analysis [53] failed to show any signal peptides or common chloroplast or mitochondrial targeting sequences amongst the eukaryote PPK1s or PPK2s. Zhao *et al.* (2008) [54] demonstrated that poly P influences intron splicing protein localisation and concentration of poly P subapically in *E. coli* and plays a crucial role in establishing cell polarity in cytokinesis. Subcellular localisation of PPK activity in eukaryotes is then also presumably important. Inorganic phosphate and smaller molecules such as ATP and GTP are highly reactive and PPK provides a mechanism for storage of phosphate in the less reactive high molecular weight poly P. For some eukaryotes, such as those adapting to novel stressful environments without the benefit of a developed vascular system, the acquisition of a bacteria type PPK and the evolution of additional *ppk* genes under the control of new promoters may provide additional opportunities for the control of poly P synthesis within the cell. A *ppk1* deletion mutant of the social slime mould *D. discoideum* had reduced levels of poly P and was deficient in development, sporulation and predation [55].

Lenton *et al.* (2012) [56], using *P. patens* as an experimental organism, have recently described the exciting hypothesis that non-vascular plants colonising rock surfaces accelerated chemical weathering, releasing phosphate for enhanced growth of oceanic phytoplankton, to the extent that falls in atmospheric carbon dioxide precipitated the global growth of ice sheets. Central to this concept is the release of phosphate to the ocean to fuel oceanic carbon fixation. Interestingly, two other eukaryotes that have bacteria type PPK1 and PPK2 are the abundant picophytoplankton *Ostreococcus tauri* and *O. lucimarinus* (Table 1[18]). In this respect, horizontal transfer of bacterial *ppk* genes to early plants such as *P. patens* colonising the land and to marine phytoplankton exploiting the consequent increase in oceanic phosphate, would have been a key factor in the slow decline in atmospheric carbon dioxide in the Ordovician.

## Conclusions

Convincing database examples of eukaryote PPKs derived from bacteria type PPK1 and PPK2 enzymes are rare and currently confined to a few simple eukaryotes. These enzymes likely represent horizontal transfer events occurring before the time of the colonisation of land by plants, with the possibility of subsequent multiple losses and further gains in different lineages. It is proposed that the retention of such horizontally transferred sequences is an advantage for stress tolerance in eukaryotes without sophisticated multicellular adaptations to stresses such as desiccation or nutrient depletion. The enhanced acquisition, release and storage of phosphates facilitated by bacterial PPKs may have promoted the colonisation of land by early plants and fuelled the growth of oceanic phytoplankton. There is very limited evidence for DNA sequences encoding PPKs in a wider range of eukaryotes, notably some invertebrates, though it is less clear that these represent functional genes.

## Methods

### Identification of PPK1 and PPK2 sequences in eukaryotes

The Interpro database (http://www.ebi.ac.uk/interpro/) was searched using keywords “polyphosphate kinase” and individual eukaryotic accessions collated. Blastp searches [19] at NCBI with the genome database (http://www.ncbi.nlm.nih.gov/sutils/genom_table.cgi) were used for direct access to individual sequences of bacterial PPK1/2 representatives by using *E. coli* PPK1 (UniProt P0A7B1) and *P. aeruginosa* PPK2 (Genbank NP_248831) as *in silico* probes. A default e-value of < 10 was used as the cut off to then determine manual scrutiny of any hits for the presence of conserved residues when screening these multiple species databases. The top matching bacterial sequence from each major bacterial grouping was chosen as the representative of that group. Some individual genomes at JGI (http://www.jgi.doe.gov/) were accessed, with a default BLASTp e-value of < 1 used to determine manual assessment of a match. *D. fasciculatum* data was accessed via the Social Amoebas Comparative Genome Browser (http://sacgb.fli-leibniz.de/cgi/index.pl?ssi=free). The *C. merolae* genome was accessed at http://merolae.biol.s.u-tokyo.ac.jp/. Phytozome v.7 (http://www.phytozome.net/) was used to screen a collection of plant genomes by Blastp for visual assessment at e < 1. The BOGAS site (http://bioinformatics.psb.ugent.be/genomes/) was used to access the *Ectocarpus silicosus* genome. The origins of multicellularity database (http://www.broadinstitute.org/annotation/genome/multicellularity_project/MultiHome.html) was used to screen some simple eukaryotes. For arthropod sequences primary amino acid sequences for *E. coli* PPK1 (Genbank NP_416996 ) and *P. aeruginosa* PPK2 (Genbank NP_248831) were used to search via Blastp, predicted proteins of the 28 completed arthropod genomes available at NCBI. The same protein sequences were then used to search genomic DNA using tBlastn to detect potential PPK encoding sequences. Any matches to accessions at lower than e-value = 0.05 were then scrutinised by EST/transcript searches at species specific databases: for *Aedes aegypti* (SRA transcripts at http://blast.ncbi.nlm.nih.gov/), *Acyrthosiphon pisum* (http://tools.genouest.org/tools/aphidblast/)*, Daphnia pulex* (http://genome.jgi-psf.org/Dappu1/Dappu1.home.html) and *Ixodes scapularis* (http://iscapularis.vectorbase.org/).

All eukaryotic sequences identified were checked for annotation within expression data and the nucleic acid sequence searched against all bacterial databases. To avoid sequence contamination all eukaryotic sequences were excluded if the level of nucleic acid identity was identical or virtually identical.

### Sequence alignment and phylogenetic analysis

Identity within different PPK1 and PPK2 proteins was observed throughout the sequences, hence entire proteins were used for the analysis. Where eukaryotic proteins had N terminal extensions, these were removed for some analyses but demonstrated very similar results as using the entire proteins. ClustalW (http://www.ebi.ac.uk/) and TCoffee [20], (http://igs-server.cnrs-mrs.fr/Tcoffee/tcoffee_cgi/index.cgi) were used to construct multiple alignments and were manually refined. Domain identification used CDD (http://www.ncbi.nlm.nih.gov/cdd) and SMART (http://smart.embl-heidelberg.de/smart/set_mode.cgi?NORMAL=1). For phylogenetic analysis both ClustalX [21] and MUSCLE [22] were used for alignment. Maximum likelihood (PhyML3.0, using the WAG model of sequence evolution) and Neighbour joining (Phylip, using the PAM 350 model of sequence evolution) analysis was viewed with Treedyn [23] or Treeview [24] and produced similar results. The presented analysis is based upon MUSCLE and Treedyn with bootstrapping values out of 100 presented (expressed as a fraction of 1). Percentage GC was calculated using http://www.genomicsplace.com/gc_calc.html. Additional file 4 provides the species used for the phylogenetic analysis.

### Availability of supporting data

Figures 2 and 3 are deposited at treebase (http://purl.org/phylo/treebase/phylows/study/TB2:S13979).

## Competing interests

The authors declare that they have no competing interests.

## Authors’ contributions

MPW carried out the phylogenetic studies and contributed to experimental design. PH was responsible for database searches, drafting of the manuscript and experimental design. MRWB conceived the study, helped with experimental design and drafting the manuscript. All authors read and approved the final manuscript.

## Supplementary Material

Additional file 1 **TCoffee multiple alignment of PPK1 from eukaryotes.** Advanced TCoffee alignment of eukaryotic PPK1s compared with prokaryotic (Uniprot: P0A7B1 *Escherichia coli*) and archaeal (Interpro: A2SQZ9 *Methanocorpusculum labreanum*) enzymes. Interpro: Q54BM7 *Dictyostelium discoideum*, Social amoebas comparative genome browser (SACGB): C300023 (DPU_G0062710) *D. purpureum*, Interpro: A2VBB6 *Porphyra yezoensis*, Interpro: Q2MEV6 *Physcomitrella patens*, Interpro: A9U2N0 *P. patens*, Interpro: Q01H21 *Ostreococcus tauri*, Interpro: A4RQI1 *O.lucimarinus*, Interpro: D3B5H9 *Polysphondylium pallidum*, Interpro: E9CFK0 *Capsaspora owczarzaki*, SACGB: EGG21828.1 *D. fasciculatum*, *C. merolae* genome database: CMM026C *Cyanidioschyzon merolae*, Interpro:F4PF87 *Batrachochytrium dendrobatidis*, Origins of multicellularity databaseAMSG11662 *Thecamonas trahens*. Highly conserved active site residues (**↑**) [18]. Click here for file

Additional file 2 **Phylogenetic analysis of PPK1 including closest matching cyanobacteria.** Numbers on the branches indicate bootstrapping values out of 100 calculated for maximum likelihood. Colours of eukaryotes indicate major taxonomic groupings (blue- non-photosynthetic eukaryotes, green- plants and green algae, red- red algae, purple-cyanobacteria). Click here for file

Additional file 3 **Phylogenetic analysis of PPK1 including weakest matching cyanobacteria.** Numbers on the branches indicate bootstrapping values out of 100 calculated for maximum likelihood. Colours of eukaryotes indicate major taxonomic groupings (blue- non-photosynthetic eukaryotes, green- plants and green algae, red- red algae, purple-cyanobacteria). Click here for file

Additional file 4 Representative species taken from bacterial taxa for phylogenetic analysis. Click here for file

## References

[B1] Rao NN Gomez-Garci MR Kornberg A Inorganic polyphosphate : essential for growth and survival Ann Rev Biochem 2009 78 605 647 19344251 10.1146/annurev.biochem.77.083007.093039

[B2] Schwartz AW Phosphorus in prebiotic chemistry Philos Trans R Soc Lond B Biol Sci 2006 361 1743 1749 17008215 10.1098/rstb.2006.1901 PMC1664685

[B3] Jones ME Lipmann F Chemical and enzymatic synthesis of carbamyl phosphate Proc Natl Acad Sci U S A 1960 46 1194 1205 16590733 10.1073/pnas.46.9.1194 PMC223023

[B4] Kulaev IS Vagabov VM Polyphosphate metabolism in micro-organisms Adv Microb Physiol 1983 24 83 171 6320606 10.1016/s0065-2911(08)60385-9

[B5] Zhang H Ishige K Kornberg A A polyphosphate kinase (PPK2) widely conserved in bacteria Proc Natl Acad Sci U S A 2002 99 16678 16683 12486232 10.1073/pnas.262655199 PMC139203

[B6] Kornberg A Kumble AD Inorganic polyphosphate in mammalian cells and tissues J Biol Chem 1995 270 5818 5822 7890711 10.1074/jbc.270.11.5818

[B7] Brown MRW Kornberg A The long and short of it – polyphosphate, PPK and bacterial survival Trends Biochem Sci 2008 33 284 290 18487048 10.1016/j.tibs.2008.04.005

[B8] Brown MRW Kornberg A Inorganic polyphosphate in the origin and survival of species Proc Natl Acad Sci U S A 2004 101 16085 16087 15520374 10.1073/pnas.0406909101 PMC528972

[B9] Zhu Y Huang W Lee SSK Xu W Crystal structure of a polyphosphate kinase and its implications for polyphosphate synthesis EMBO Rep 2005 6 681 687 15947782 10.1038/sj.embor.7400448 PMC1369109

[B10] Shiba T Itoh H Kameda A Kobayashi K Kawazoe Y Noguchi T Polyphosphate:AMP phosphotransferase as a polyphosphate-dependent nucleoside monophosphate kinase in *Acinetobacter johnsonii* 210A J Bact 2005 187 1859 1865 15716459 10.1128/JB.187.5.1859-1865.2005 PMC1063994

[B11] Nocek B Kochinyan S Proudfoot M Brown G Evdokimova E Osipiuk J Edwards AM Savchenko A Joachimiak A Yakunin AF Polyphosphate-dependent synthesis of ATP and ADP by the family-2 polyphosphate kinases in bacteria Proc Natl Acad Sci U S A 2008 105 17730 17735 19001261 10.1073/pnas.0807563105 PMC2584756

[B12] Kuroda A Murphy H Cashel M Kornberg A Guanosine tetra- and pentaphosphate promote accumulation of inorganic polyphosphate in *Escherichia coli* J Biol Chem 1997 272 21240 21243 9261133 10.1074/jbc.272.34.21240

[B13] Gomez-Garcia MR Kornberg A Formation of an actin like filament concurrent with the enzymatic synthesis of inorganic polyphosphate Proc Natl Acad Sci U S A 2004 101 15876 15880 15496465 10.1073/pnas.0406923101 PMC528760

[B14] Hothorn M Neumann H Lenherr ED Wehner M Rybin V Hassa PO Uttenweiler A Reinhardt M Schmidt A Seiler J Ladurner AG Herrmann C Scheffzek K Mayer A Catalytic core of a membrane-associated eukaryotic polyphosphate polymerase Science 2009 324 513 516 19390046 10.1126/science.1168120

[B15] Reusch RN Huang R Koch-Kosuka D Novel components and enzymatic activities of the human erythrocyte plasma membrane pump FEBS Letts 1997 412 592 596 9276473 10.1016/s0014-5793(97)00863-6

[B16] Lonetti A Szijgyarto Z Bosch D Loss O Azevedo C Saiardi A Identification of an evolutionarily conserved family of inorganic polyphosphate endopolyphosphatases J Biol Chem 2011 286 37 31966 31974 21775424 10.1074/jbc.M111.266320 PMC3173201

[B17] Schaack S Gilbert C Fechotte C Promiscuous DNA: horizontal transfer of transposable elements and why it matters for eukaryotic evolution Trends Ecol Evol 2010 25 537 546 20591532 10.1016/j.tree.2010.06.001 PMC2940939

[B18] Hooley P Whitehead MP Brown MRW Eukaryote polyphosphate kinases – is the “Kornberg” complex ubiquitous? Trends Biochem Sci 2008 33 577 582 18938082 10.1016/j.tibs.2008.09.007

[B19] Altschul SF Madden TL Schäffer AA Zhang J Zhang Z Miller W Lipman DJ Gapped blast and psi-blast: a new generation of protein database search programs Nucleic Acids Res 1997 25 3389 3402 9254694 10.1093/nar/25.17.3389 PMC146917

[B20] Notre-Dame C Higgins DG Heringa J T-Coffee: A novel method for multiple sequence alignments J Mol Biol 2000 302 205 217 10964570 10.1006/jmbi.2000.4042

[B21] Thompson JD Gibson TJ Plewniak F Jeanmougin F Higgins DG The CLUSTAL-X windows interface: flexible strategies for multiple sequence alignment aided by quality analysis tools Nucleic Acids Res 1997 25 4876 4882 9396791 10.1093/nar/25.24.4876 PMC147148

[B22] Edgar RC MUSCLE: multiple sequence alignment with high accuracy and high throughput Nucleic Acids Res 2004 32 1792 1797 15034147 10.1093/nar/gkh340 PMC390337

[B23] Dereeper A Guignon V Blanc G Audic S Buffet S Chevenet F Dufayard JF Guindon S Lefort V Lescot M Claverie JM Gascuel O Phylogeny.fr: robust phylogenetic analysis for the non-specialist Nucleic Acids Res 2008 36 W465 W469 18424797 10.1093/nar/gkn180 PMC2447785

[B24] Page RMD Treeview. An application to display phylogenetic trees on personal computers Comp Appl Biosci 1996 12 357 358 8902363 10.1093/bioinformatics/12.4.357

[B25] Eichinger LL Noegel AA Crawling into a new era – the *Dictyostelium* genome project EMBO J 2003 22 1941 1946 12727861 10.1093/emboj/cdg214 PMC156086

[B26] Kornberg A Abundant microbial inorganic polyphosphate, Poly P kinases are underappreciated Microbe 2008 3 119 123

[B27] Lang D Zimmer AD Rensing SA Resk R Exploring plant biodiversity : the *Physcomitrella* genome and beyond Trends Plant Sci 2008 13 542 549 18762443 10.1016/j.tplants.2008.07.002

[B28] Poptsova MS Gogarten JP Using comparative genome analysis to identify problems in annotated microbial genomes Microbiology 2010 156 1909 1917 20430813 10.1099/mic.0.033811-0

[B29] Dunning Hotopp JC Clark ME Oliveira DC Foster JM Fischer P Muñoz Torres MC Giebel JD Kumar N Ishmael N Wang S Ingram J Nene RV Shepard J Tomkins J Richards S Spiro DJ Ghedin E Slatko BE Tettelin H Werren JH Widespread lateral gene transfer from intracellular bacteria to multicellular eukaryotes Science 2007 317 1753 1756 17761848 10.1126/science.1142490

[B30] Nikoh N Tanaka K Shibata F Kondo N Hizume M Shimada M Fukatsu T *Wolbachia* genome integrated in an insect chromosome: evolution and fate of laterally transferred endosymbiont genes Genome Res 2008 18 272 280 18073380 10.1101/gr.7144908 PMC2203625

[B31] Klasson L Kambris Z Cook PE Walker T Sinkins SP Horizontal gene transfer between *Wolbachia* and the mosquito *Aedes aegypti* BMC Genomics 2009 10 33 10.1186/1471-2164-10-33 19154594 PMC2647948

[B32] Finazzi G Moreau H Bowler C Genomic insights into photosynthesis in eukaryotic phytoplankton Trends Plant Sci 2010 15 565 572 20800533 10.1016/j.tplants.2010.07.004

[B33] Huang J Gogarten JP Concerted gene recruitment in early plant evolution Genome Biol 2008 9 R109 http://genomebiology.com/2008/9/7/R109 18611267 10.1186/gb-2008-9-7-r109 PMC2530860

[B34] Derelle E Ferraz C Escande ML Eychenié S Cooke R Piganeau G Desdevises Y Bellec L Moreau H Grimsley N Life cycle and genome of OtV5, a large DNA virus of the pelagic marine unicellular green alga *Ostreococcus tauri* PLoS One 2008 3 e2250 10.1371/journal.pone.0002250 18509524 PMC2386258

[B35] Raymond J Blankenship RE Horizontal gene transfer in eukaryotic algal evolution Proc Natl Acad Sci U S A 2003 100 7419 7420 12810941 10.1073/pnas.1533212100 PMC164597

[B36] Rohwer F Thurber RV Viruses manipulate the marine environment Nature 2009 459 207 212 19444207 10.1038/nature08060

[B37] Richards TA Genome evolution:horizontal movement in the fungi Curr Biol 2011 21 R166 21334300 10.1016/j.cub.2011.01.028

[B38] Roy SW Irimia M Mystery of intron gain: new data and new models Trends Genet 2009 25 2 67 73 19070397 10.1016/j.tig.2008.11.004

[B39] Jeffares DC Mourier T Penny D The biology of intron gain and loss Trends Genet 2006 22 16 22 16290250 10.1016/j.tig.2005.10.006

[B40] Gladyshev EA Meselson M Arkhipova IR Massive horizontal gene transfer in bdelloid rotifers Science 2008 320 1210 1213 18511688 10.1126/science.1156407

[B41] Roy SW Irimia M Penny D Very little intron gain in *Entamoeba histolytica* genes laterally transferred from prokaryotes Mol Biol Evol 2006 23 1824 1827 16847043 10.1093/molbev/msl061

[B42] Roy SW Penny D Patterns of intron loss and gain in plants: intron-loss dominated evolution and genome-wide comparison of *O. sativa* and *A.thaliana* Mol Biol Evol 2007 24 171 181 17065597 10.1093/molbev/msl159

[B43] Csuros M Rogozin IB Koonin EV A detailed history of intron-rich eukaryotic ancestors inferred from a global survey of 100 complete genomes PLoS Comp. Biol 2011 7 e1002150 10.1371/journal.pcbi.1002150 PMC3174169 21935348

[B44] Gustavsson S Lebrun A-S Nordén K Chaumont F Johanson U A novel plant major intrinsic protein in *Physcomitrella patens* most similar to bacterial glycerol channels Plant Physiol 2003 139 287 295 16113222 10.1104/pp.105.063198 PMC1203378

[B45] Stenoien HK Compact genes are highly expressed in the moss *Physcomitrella patens* J Evol Biol 2007 20 1223 1229 17465932 10.1111/j.1420-9101.2007.01301.x

[B46] Markmann-Mulisch U Hadi MZ Koepchen K Alonso JC Russo VE Schell J Reiss B The organization of *Physcomitrella patens RAD51* genes is unique among eukaryotic organisms Proc Natl Acad Sci U S A 2002 99 2959 2964 11880641 10.1073/pnas.032668199 PMC122455

[B47] Sucgang R Kuo A Tian X Salerno W Parikh A Feasley CL Dalin E Tu H Huang E Barry K Lindquist E Shapiro H Bruce D Schmutz J Salamov A Fey P Gaudet P Anjard C Babu MM Basu S Bushmanova Y van der Wel H Katoh-Kurasawa M Dinh C Coutinho PM Saito T Elias M Schaap P Kay RR Henrissat B Eichinger L Rivero F Putnam NH West CM Loomis WF Chisholm RL Shaulsky G Strassmann JE Queller DC Kuspa A Grigoriev IV Comparative genomics of the social amoebae *Dictyostelium discoideum* and *Dictyostelium purpureum* Genome Biol 2011 12 R20 21356102 10.1186/gb-2011-12-2-r20 PMC3188802

[B48] Hirano K Nakajima M Asano K Nishiyama T Sakakibara H Kojima M Katoh E Xiang H Tanahashi T Hasebe M Banks JA Ashikari M Kitano H Ueguchi-Tanaka M Matsuoka M The GID-1 mediated gibberellin perception mechanism is conserved in the lycophyte *Selaginella meoellendorffii* but not in the bryophyte *Physcomitrella patens* Plant Cell 2007 19 3058 3079 17965273 10.1105/tpc.107.051524 PMC2174699

[B49] Yasigawa F Nishida K Yoshida M Ohnuma M Shimada T Fujiwara T Yoshida Y Misumi O Kuroiwa H Kuroiwa T Identification of novel proteins in isolated polyphosphate vacuoles in the primitive red alga *Cyanidioschyzon merolae* Plant J 2009 60 882 893 19709388 10.1111/j.1365-313X.2009.04008.x

[B50] Mitsuhashi N Ohnishi M Sekiguchi Y Kwon YU Chang YT Chung SK Inoue Y Reid RJ Yagisawa H Mimura T Phytic acid synthesis and vacuolar accumulation in suspension-cultured cells of *Catharanthus roseus* induced by high concentration of inorganic phosphate and cations Plant Physiol 2005 138 1607 1614 15965017 10.1104/pp.105.060269 PMC1176430

[B51] Le Bihan T Martin SF Chirnside ES Van Ooijen G Barrios-Llerena ME O’Neill JS Shliaha PV Kerr LE Millar AJ Shotgun proteomic analysis of the unicellular alga *Ostreococcus tauri* J Proteomics 2011 74 2060 2070 21635980 10.1016/j.jprot.2011.05.028

[B52] Nishiyama T Fujita T Shin-I T Seki M Nishide H Uchiyama I Kamiya A Carninci P Hayashizaki Y Shinozaki K Kohara Y Hasebe M Comparative genomics of *Physcomitrella patens* gametophytic transcriptome and *Arabidopsis thaliana*; implication for land plant evolution Proc Natl Acad Sci U S A 2003 100 8007 8012 12808149 10.1073/pnas.0932694100 PMC164703

[B53] Emanuelsson O Brunak S Von Heijne G Nielson H Locating proteins in the cell using TargetP, SignalP and related tools Nature Protoc 2007 2 953 971 17446895 10.1038/nprot.2007.131

[B54] Zhao J Zhao J Niu W Yao J Mohr S Marcotte EM Lambowitz AM Group II intron protein localisation insertion sites are affected by polyphosphate PLoS Biol 2008 6 e150 http://www.plosbiology.org/article/info%3Adoi%2F10.1371%2Fjournal.pbio.0060150 18593213 10.1371/journal.pbio.0060150 PMC2435150

[B55] Zhang H Gomez-Garcia MR Brown MRW Kornberg A Inorganic polyphosphate in *Dictyostelium*: influence on development, sporulation and predation Proc Natl Acad Sci U S A 2005 102 2731 2735 15701689 10.1073/pnas.0500023102 PMC549442

[B56] Lenton TM Crouch M Johnson M Pires N Dolan L First plants cooled the Ordovician Nature Geosci 2012 5 86 89

