# Supplementary material for: Horizontal transfer of bacterial polyphosphate kinases to eukaryotes: implications for the ice age and land colonisation
Source: BMC Res Notes. 2013 Jun 5;6:221. doi: 10.1186/1756-0500-6-221 (PMC3680246; doi:10.1186/1756-0500-6-221)
Supplement: Additional file 1 — TCoffee multiple alignment of PPK1 from eukaryotes. Advanced TCoffee alignment of eukaryotic PPK1s compared with prokaryotic (Uniprot: P0A7B1 Escherichia coli) and archaeal (Interpro: A2SQZ9 Methanocorpusculum labreanum) enzymes. Interpro: Q54BM7 Dictyostelium discoideum, Social amoebas comparative genome browser (SACGB): C300023 (DPU_G0062710) D. purpureum, Interpro: A2VBB6 Porphyra yezoensis, Interpro: Q2MEV6 Physcomitrella patens, Interpro: A9U2N0 P. patens, Interpro: Q01H21 Ostreococcus tauri, Interpro: A4RQI1 O.lucimarinus, Interpro: D3B5H9 Polysphondylium pallidum, Interpro: E9CFK0 Capsaspora owczarzaki, SACGB: EGG21828.1 D. fasciculatum, C. merolae genome database: CMM026C Cyanidioschyzon merolae, Interpro:F4PF87 Batrachochytrium dendrobatidis, Origins of multicellularity databaseAMSG11662 Thecamonas trahens. Highly conserved active site residues (↑) [18]. [file 1756-0500-6-221-S1.pdf]

Evidence for horizontal transfer of bacterial polyphosphate kinases in a small group of diverse, simple eukaryotes.

Whitehead, M.P., Hooley P. and Brown M.R.W.

Additional File 1. TCoffee multiple alignment of PPK1 from eukaryotes.
 Advanced TCoffee alignment of eukaryotic PPK1s compared with prokaryotic (Uniprot: P0A7B1 *Escherichia coli*) and archaeal (Interpro: A2SQZ9 *Methanocorpusculum labreanum*) enzymes. Interpro: Q54BM7 *Dictyostelium discoideum*, Social amoebas comparative genome browser (SACGB): C300023 (DPU_G0062710) *D. purpureum*, Interpro: A2VBB6 *Porphyra yezoensis*, Interpro: Q2MEV6 *Physcomitrella patens*, Interpro: A9U2N0 *P. patens*, Interpro: Q01H21 *Ostreococcus tauri*, Interpro: A4RQI1 *O.lucimarinus*, Interpro: D3B5H9 *Polysphondylium pallidum*, Interpro: E9CFK0 *Capsaspora owczarzaki*, SACGB: EGG21828.1 *D. fasciculatum*, *Cyanidioschyzon merolae* genome database: CMM026C *Cyanidioschyzon merolae*, Interpro:F4PF87 *Batrachochytrium dendrobatidis*, Origins of multicellularity database AMSG11662 *Thecamonas trahens*. Highly conserved active site residues (↑) [18].

```
  BAD AVG GOOD
*
Q54BM7       :  79
C300023      :  81
P0A7B1       :  82
A2VBB6       :  81
Q2MEV6       :  80
A9U2N0       :  81
Q01H21       :  83
A4RQI1       :  84
A2SQZ9       :  81
D3B5H9       :  77
E9CFK0       :  64
EGG21828.1   :  77
CMM026C      :  75
F4PF87       :  84
AMSG11662    :  74
cons         :  79

Q54BM7          1 MITNSKMENKI-LDEFDEE----------------------------E   19
C300023         1 MELHRYKISQ--------------------------------------   10
P0A7B1          1 MGQ---------------------------------------------    3
A2VBB6          1 GGSG--------------------------------------------    4
Q2MEV6          1 MEGAAAGLGSTSVGR----------------SSQPGRS-YLFCH----   27
A9U2N0          1 MQSVASE-GASCSGP----------------GPQPGDG----------   21
Q01H21          1 MDSETAGAKMDANSL---------------------------------   15
A4RQI1          1 MEEDKD------------------------------------------    6
A2SQZ9          1 MGKIQKSGKTDK------------------------------------   12
D3B5H9          1 MDNKTTPDDKNVSSEYSNDFKHIMKSSLNVENQKTENISPFKS-IDTT   47
E9CFK0          1 MVTEASDQPDSQ---------------------------PPA----L-   16
EGG21828.1      1 MTMAKNIPI-TSD-----------------------NDSPIVA-----   19
CMM026C         1 MRCCELGPNTGA--------------------DQPERTEPPS----ET   24
F4PF87          1 MVR---------------------------------------------    3
AMSG11662       1 MPHRKRKARPMLPT----P-----QVGLNVNDIMPHGRTPPSPHI-TP   38

cons            1                                                    48


Q54BM7         20 LNKLKINSNNKESTTTTTSTTTTTTTT---------------------   46
C300023        11 ------------------------------------------------   10
P0A7B1          4 ------------------------------------------------    3
A2VBB6          5 ------------------------------------------------    4
Q2MEV6         28 -----IESNARSSA----LLAG--------------------------   40
A9U2N0         22 -----LESGE--------------------------------------   26
Q01H21         16 ------------------------------------------------   15
A4RQI1          7 ------------------------------------------------    6
A2SQZ9         13 ------------------------------------------------   12
```

```
D3B5H9        48 VSSDSINSEA--SNGTNTSTTTTTSTTDSANNL-AAIINDDLNRRLVE  92
E9CFK0        17 ------------------------------------------------  16
EGG21828.1    20 ----------------TTQQLKELSVGDIDDDKPSIELDDNDGREPLL  51
CMM026C       25 -----SESDEVAAA----VAAAAAAAGDLADRL---------T-----  49
F4PF87         4 ------------------------------------------------   3
AMSG11662     39 -----VDRDDAGSA----PVALASRMQAPP--H---------A-----  61

cons          49                                                   96


Q54BM7        47 ----------------------------TSTSGEESDDEHTG-SSSST  65
C300023       11 ------------------------------------------------  10
P0A7B1         4 ------------------------------------------------   3
A2VBB6         5 ------------------------GGV---------------------   7
Q2MEV6        41 -------------------------------------------STAMA  45
A9U2N0        27 ------------------------------------------------  26
Q01H21        16 ------------------------------------------------  15
A4RQI1         7 ------------------------------------------------   6
A2SQZ9        13 ------------------------------------------------  12
D3B5H9        93 DASSVNHIGNSINHLSVNDNTNHRENRVSPQSSIDEEITQFMNHNGNG 140
E9CFK0        17 ------------------------------------------------  16
EGG21828.1    52 PVHSFVHGNNNNNNQQLDTPTSSTNGVVTSSANEDEEVIHLL-HNHNK  98
CMM026C       50 ------------------------RSV---------------------  52
F4PF87         4 ------------------------------------------------   3
AMSG11662     62 ------------------QPAASTNGT---------------------  70

cons          97                                                  144


Q54BM7        66 AAMEL--HRYKISQECPVDFEDDYDEESS----SFD--EEDE-D---S 101
C300023       11 --------------ECPVDFEDDYDEESS----SFY--EEDE-D---S  34
P0A7B1         4 ------------------------------------------------   3
A2VBB6         8 -----------------------------------G--DGI--D---A  13
Q2MEV6        46 CGIKC--HH-------------WQNRVTS----SE-------------  61
A9U2N0        27 ------------------------------------------------  26
Q01H21        16 ------------------------------------------------  15
A4RQI1         7 ------------------------------------------------   6
A2SQZ9        13 ------------------------------------------------  12
D3B5H9       141 NHHHHHHHSNHDDDD------DYHHENGGSVKTEESDIESDDEKTTQ- 181
E9CFK0        17 ------------------------------------------------  16
EGG21828.1    99 IESDV--HSSLDSDDERTNQEDEYSEDGS----EEDRYETDDSDDTDS 140
CMM026C       53 -----------------------------------D--EQSR-S---A  59
F4PF87         4 ------------------------------------------------   3
AMSG11662     71 -----------------------------------G--DSGR-P---A  77

cons         145                                                  192


Q54BM7       102 -------------------------------------------AA-ES 105
C300023       35 -------------------------------------------SA-QE  38
P0A7B1         4 ------------------------------------------------   3
A2VBB6        14 ---------GGGSGGGG-DPPLP--PPSVTPLLPPPAPPLSSASSPLS  49
Q2MEV6        62 ------------------------------------------------  61
A9U2N0        27 ------------------------------------------------  26
Q01H21        16 ------------------------------------------------  15
A4RQI1         7 ------------------------------------------------   6
A2SQZ9        13 ------------------------------------------------  12
D3B5H9       182 --PSFSSPCVTNESMN--------------DYEDA-----STDSSDLS 208
E9CFK0        17 ------------------------------------------------  16
EGG21828.1   141 DVDYFGMDALTNSSSNG-R--LS--HPHMAPSSPS-----SSSSL-SS 177
CMM026C       60 ---------GCAASGAQD-KPQT--LCSVQ-DVT--------ASTAQR  86
F4PF87         4 ------------------------------------------------   3
AMSG11662     78 ---------TVARPGSQQHPPLPASHPSAVPDTPP-----STSSTPLG 111

cons         193                                                  240


Q54BM7       106 NGG-------NGKLIPNPND------------------K-KKKRSSK- 126
```

```
C300023        39 NP---------ANLIPNPNDKKIKP---------RIGNK-KKKESTT-   66
P0A7B1          4 ------------------------------------------------   3
A2VBB6         50 -----------G------------------------------------   50
Q2MEV6         62 -------------VISV------------ASGVRSIARKM-QQRCFKK   83
A9U2N0         27 ------------------------------------------------   26
Q01H21         16 ------------------------------------------------   15
A4RQI1          7 ------------------------------------------------   6
A2SQZ9         13 ------------------------------------------------   12
D3B5H9        209 DS---------DN----YDNTNAIPRKVINSNMPKRSSKE-KERLLKL   242
E9CFK0         17 ------------------------------------------------   16
EGG21828.1    178 ST---------SNLPPAPHHTLPTPSSMINHNAQRKSKKMNKKKSSKS   216
CMM026C        87 --FDVRQSKECG------EAVQNFSELGV-------------------   107
F4PF87          4 ------------------------------------------------   3
AMSG11662     112 --VDAKTGPSNG------KQSHAFPTSLVSSTV---------------   136

cons          241                                                    288


Q54BM7        127 ------------------SSSKS---------SKSKSKSKNKETTANP   147
C300023        67 ------------------TSTTNNE-FPS---KESIDKLKSKGPIQNP   92
P0A7B1          4 ------------------------------------------------   3
A2VBB6         51 ------------------------------------------------   50
Q2MEV6         84 GAAGEAFSNISSQLGCGRESLERPRSQTT-RLAKKKQNAWSRGGVRVR   130
A9U2N0         27 ------------------------------------------------   26
Q01H21         16 ------------------------------------------------   15
A4RQI1          7 ------------------------------------------------   6
A2SQZ9         13 ------------------------------------------------   12
D3B5H9        243 NG----GGNHSGGSSGSNASKSKKKHLSFKDKLKEREKEKEKDKDHHK   286
E9CFK0         17 ------------------------------------------------   16
EGG21828.1    217 NN----NNNTTSTSSTTTTTTTNNNSSST-RSKKDKDKKKEKGKGKSK   259
CMM026C       108 ---------------------------------------GKQQ-----   111
F4PF87          4 ------------------------------------------------   3
AMSG11662     137 ---------------------------------------HDKE-----   140

cons          289                                                    336


Q54BM7        148 V-------------QNCQLME-----------------SGT--LFSPT   163
C300023        93 L------------VKSQVLQESNN--------------QSG--LFSPT   112
P0A7B1          4 ------------------------------------------------   3
A2VBB6         51 ------------------------------------------------   50
Q2MEV6        131 -ADAVVQQSAGSDETTGNRLVENGVATLVTLKAVIDEKSGA--LLLKE   175
A9U2N0         27 ---------TGRESLNGDGFSEDGAST-VNLLGGFDLKFGT--LVLPE   62
Q01H21         16 ------------------------------------------------   15
A4RQI1          7 ------------------------------------------------   6
A2SQZ9         13 ------------------------------------------------   12
D3B5H9        287 -GDASTTEHFAHDKKLSSLRAECG--------------QSP--LFSPT   317
E9CFK0         17 ------------------------------------------------   16
EGG21828.1    260 SMDGRDSLDLGKDKKIHSLIRSEA--------------NSP--LFSPT   291
CMM026C       112 ----------------------E-------------------------   112
F4PF87          4 ------------------------------------------------   3
AMSG11662     141 ----------------------DT--------------PSAVAIASP-   151

cons          337                                                    384


Q54BM7        164 EFVKSST-----VN--------PS-----KSPVQP--IKL--------   183
C300023       113 EFVKSST-----VS--------QA-----KSPA-P--IKL--------   131
P0A7B1          4 ------------------------------------------------   3
A2VBB6         51 ------------------------------------------------   50
Q2MEV6        176 ELTKEK------------------------------------------   181
A9U2N0         63 EGLKKQ------------------------------------------   68
Q01H21         16 ------------------------------------------------   15
A4RQI1          7 ------------------------------------------------   6
A2SQZ9         13 ------------------------------------------------   12
D3B5H9        318 EFVKSST-----NN--------IQ-----PTVGTQIHNLT--------   339
E9CFK0         17 ------------------------------------------------   16
EGG21828.1    292 EFVKGAGGGSSSHQHHH-----HP-----PQPLTTQHNLS--------   321
```

```
CMM026C       113 -HADPRT-----VW--------LP-----GSPE-P--TAFPQVRLVGN 138
F4PF87          4 ------------------------------------------------   3
AMSG11662     152 MIAKPST-----RF-SISPSATLPTSVMPAVP--S--LPADK-HQQNQ 188

cons          385                                                  432


Q54BM7        184 --SSMLGLSELGTSNVLT---VQNPQPISEPLKNDISSGSSSS----- 221
C300023       132 --SSMLGLSSLGTSNLLS---VQNPQPIEG------------------ 156
P0A7B1          4 ------------------------------------------------   3
A2VBB6         51 EDHPP-------------------------------------------  55
Q2MEV6        182 ND---------------------------------------------- 183
A9U2N0         69 DD----------------------------------------------  70
Q01H21         16 ------------------------------------------------  15
A4RQI1          7 ------------------------------------------------   6
A2SQZ9         13 ------------------------------------------------  12
D3B5H9        340 --SSLMGFRNLGASNLLS---VQNPQVIPSSN---------------- 366
E9CFK0         17 ------------------------------------------------  16
EGG21828.1    322 --SSLMGFRNLGCSNILQ---NQNPQIIPSGS---------------- 348
CMM026C       139 WDLPMVPMNEEPSEYTSSVREGAVPDLCEEGSV-TRASRIGACVVQAA 185
F4PF87          4 ------------------------------------------------   3
AMSG11662     189 KDTPPAKTARHPTDDPAL---VATPEPASAAAA-TTTAG--------- 223

cons          433                                                  480


Q54BM7        222 ----------------------SSSNNNNSNSNSNGNCNSDSTKSKFT 247
C300023       157 ---------------------------------------------TQG 159
P0A7B1          4 ------------------------------------------------   3
A2VBB6         56 ------------------------------------------------  55
Q2MEV6        184 ------------------------------------------------ 183
A9U2N0         71 ------------------------------------------------  70
Q01H21         16 ------------------------------------------------  15
A4RQI1          7 ------------------------------------------------   6
A2SQZ9         13 ------------------------------------------------  12
D3B5H9        367 -------------------------------------------NLPSN 371
E9CFK0         17 ------------------------------------------------  16
EGG21828.1    349 -------------------------------------------KPLLT 353
CMM026C       186 SHSREAVAGDAQSTDDQESEMHTAS-ASTTTASSLKQCTADGKSGEST 232
F4PF87          4 ------------------------------------------------   3
AMSG11662     224 ------------------------------------------------ 223

cons          481                                                  528


Q54BM7        248 SLNSWDAPPLAN---VLPDPSYCQDIVDSENN-FIVP--SVKHTILDV 289
C300023       160 TLNNWDAPPLHT---VLPDPSYCQDTIDDEDN-FIVP--TSKYSFLDI 201
P0A7B1          4 ------------------------------------------------   3
A2VBB6         56 --LSFGIPSAAA---VLPPPA--DQL---H------------------  75
Q2MEV6        184 --------------------------TTHAS----------------- 188
A9U2N0         71 --------------------------TAQASN-FKLSVVYAEEEVCDY  91
Q01H21         16 ------------------------------------------------  15
A4RQI1          7 ------------------------------------------------   6
A2SQZ9         13 ------------------------------------------------  12
D3B5H9        372 PMVFSIAPPVGN---TLPDSDYLQKVQTDDDH-FIVP--CCNYPILDT 413
E9CFK0         17 ------------------------------------------------  16
EGG21828.1    354 TLMGSGDPNLGR---VLPDPEYLPHVWDEQDNVFIVP--TCSHPILDI 396
CMM026C       233 QRLHTDSRASSG-IAFVPYKSEEEVLADLEL-EDSMD--DHTYTLLDL 276
F4PF87          4 ------------------------------------------------   3
AMSG11662     224 PAATAAAPPTSTQAATPPSPSPGDDA---EP--DSAP--A-------S 257

cons          529                                                  576


Q54BM7        290 DNLFNNLIQN-T----------NNNN--------------NND-FKSS 311
C300023       202 NNLFSSNYFMGS--NNCLTTPTNSDT--------------NSHFIKNS 233
P0A7B1          4 ------------------------------------------------   3
A2VBB6         76 ------------------------------------------------  75
```

```
Q2MEV6         189  -----------------KSKLRKLK---------------PSKIDDLP  204
A9U2N0          92  AYSFSFTRLT--QVSDTKPKPRKSR---------------ASKIVEVS  122
Q01H21          16  -----------------------------------------------R   16
A4RQI1           7  ------------------------------------------------    6
A2SQZ9          13  ------------------------------------------------   12
D3B5H9         414  ENLFTDIVLPQTNPGNYLSTPSGGTPRLVPSASNSDI-GQPT-----S  455
E9CFK0          17  ------------------------------------------------   16
EGG21828.1     397  DNLFSDVSVPA---GGYPSTPGDSTPRLLQSQSVGDILSHPSHTSLQS  441
CMM026C        277  RKSLPEQMQR--------------------------------------  286
F4PF87           4  ------------------------------------------------    3
AMSG11662      258  AMSTPPGSSA---------------G-----------TSRPSHAPGHS  279

cons           577                                                    624

Q54BM7         312  TMIGKPFSSGGDGSTSP---LI----SGLSSSSI------IPNG---N  343
C300023        234  GKINDPTSSTGT---------------------V------TP------  248
P0A7B1           4  ------------------------------------------------    3
A2VBB6          76  ------------------------------------------------   75
Q2MEV6         205  VELRQPPMNG--------------------------------------  214
A9U2N0         123  DELGEPYALG--------------------------------------  132
Q01H21          17  ELFNESIAVI--------------------------------------   26
A4RQI1           7  ------------------------------------------------    6
A2SQZ9          13  ------------------------------------------------   12
D3B5H9         456  YHLQPPSSSSSD-YTKT---NGLASSSHLPKSQL------KPTNNNEE  493
E9CFK0          17  ------------------------------------------------   16
EGG21828.1     442  TTLNHPSMASSY-YQMP---NNAG-GSSLNNSTA------IPKYFNSF  478
CMM026C        287  ----------RS-YACPTVRDGSRSDRHLDRRAFRGENEEEHDDDSSS  323
F4PF87           4  ------------------------------------------------    3
AMSG11662      280  G-LGA-----SA-AAATTAAAAAAASSKAVR--FSVD--DDSDDSDE-  315

cons           625                                                    672

Q54BM7         344  GS--L-AEQ---QQQQQ--------QS--I--PESEIPIDIKVLQLSG  373
C300023        249  ------------PFSNN--------EP--I--SESEIPLDVKVMQLSG  272
P0A7B1           4  ------------------------------------------------    3
A2VBB6          76  --RYQ-VCP---PTNHY-----TPPV-------APGQLEALSNEELSS  105
Q2MEV6         215  -------------------NVVMPPQKQEGL--EAD-VAGIGVDTLVA  240
A9U2N0         133  -------------------NVVMPLQKQEGL--EAD-VADIGLDALVV  158
Q01H21          27  -------------------RTVAPPFAQVGTEESRDVLKELDVETLTR   55
A4RQI1           7  ------------------------------------ALSEYDVETLVK   18
A2SQZ9          13  -----------------------------------NKYLKKSTRFPKE   25
D3B5H9         494  -T--T-TTT---NN-NN--------E--ND--LTEDLPMDLKILQLSG  521
E9CFK0          17  -------------------------------------------QSPAA   21
EGG21828.1     479  GT--T-SEQ---QQQQQ--------QQVPG--MDNDLPLDIKILQLSG  510
CMM026C        324  GPDRDVWTPPATPLDSEMPLIVCPPEPVWP---CEEDLRDLTPAELAR  368
F4PF87           4  ------------------------------------------------    3
AMSG11662      316  -EEAD-YYN---PFNKE-P-I-VLPRVGMG---RMEQVLDMSIAELAS  352

cons           673                                                    720

Q54BM7         374  SRMFFNRE-----------LSELIYFYRILYEAYNP------------  398
C300023        273  SRLFFNRE-----------LSELIYFYRILYEAYNT------------  297
P0A7B1           4  EKLYIEKE-----------LSWLSFNERVLQEAADK------------   28
A2VBB6         106  SELVFSKE-----------FAELSFNSRVLALALDD------------  130
Q2MEV6         241  ADSVYNKE-----------LSWLAFNWRVLHMALDV------------  265
A9U2N0         159  ADLVYNKELRVSANTFSLVCSWLAFNWRVLHMALDV------------  194
Q01H21          56  SDSVFNPE-----------MSWLAFNWRVLAMAANE------------   80
A4RQI1          19  SDPVFNAE-----------MSWLAFNWRVLAMAANE------------   43
A2SQZ9          26  SGRYFNRE-----------LSWLKFNERVLYEAENT------------   50
D3B5H9         522  SRIYFNRE-----------LTELIYFYRILYEGYNQ------------  546
E9CFK0          22  NCMRFSRR-----------DAELAFLDRVLDLADTTAAAAAVLDGDAR   58
EGG21828.1     511  SRHYFNRE-----------LTELIYFYRILYEAYNP------------  535
CMM026C        369  STDVFMQV-----------AEELHFHWRVLKLGTDP------------  393
F4PF87           4  ------------------------------------------------    3
AMSG11662      353  SPLVFNRE-----------LSELDFFWRVLYEAMDE------------  377
```

```
cons          721                                                    768

Q54BM7        399 ------------------------------------TYPILERVRFIA 410
C300023       298 ------------------------------------TYPILERVRFIA 309
P0A7B1         29 ------------------------------------SNPLIERMRFLG  40
A2VBB6        131 ------------------------------------KLPLLERFKFCC 142
Q2MEV6        266 ------------------------------------RTPLLERLRFLA 277
A9U2N0        195 ------------------------------------HVPLFERLRFLA 206
Q01H21         81 ------------------------------------DTPIFERLRFVA  92
A4RQI1         44 ------------------------------------EVPIFERLRFVA  55
A2SQZ9         51 ------------------------------------DNPLLERVTFLG  62
D3B5H9        547 ------------------------------------AAPVLEKIRFLA 558
E9CFK0         59 PKPRRAHGQGDAVETPTPTPAASSLEHWSGGGGGGECIPLLERLRFLA 106
EGG21828.1    536 ------------------------------------AVPVIEKLRFLA 547
CMM026C       394 ----------------------------------VTPTPLLERLKFLC 407
F4PF87          4 ------------------------------------------------   3
AMSG11662     378 ------------------------------------KAPLLERIKFLA 389

cons          769                                                    816

Q54BM7        411 ITSQNLDMYFCKRALKLRLGYI-------------------------- 432
C300023       310 ITSQNLDMYFCKRALKLRLGYI-------------------------- 331
P0A7B1         41 IYSNNLDEFYKVRFAELKRRIIISEE----------------------  66
A2VBB6        143 IVSANLDEHFAKRLGNIPRDDSDDEDGDGSTPAPAVSSAASSGSANSL 190
Q2MEV6        278 ISARNLDEFFCKRIGALKRQEAAGVE---------------------- 303
A9U2N0        207 ISACNIDEFFCKRVGALKRQEAAGVE---------------------- 232
Q01H21         93 ISARNLDEFFAKRVGAIKRQEEAGVE---------------------- 118
A4RQI1         56 ISARNLDEFFAKRVGAIKRQAAAGLE----------------------  81
A2SQZ9         63 IVAGNLDEFFMVRVPAYQKGATYSAD----------------------  88
D3B5H9        559 ITSQNLDMYFCKRALKLRLGYI-------------------------- 580
E9CFK0        107 IVASSLDAFSLKRLGKLELEQSQANAA--------------------V 134
EGG21828.1    548 ITSQNIDMYFCKRALKLRLGFI-------------------------- 569
CMM026C       408 IVSSNLDEYFSKNMYDVSKTMRPL------------------------ 431
F4PF87          4 -------------VAGLKDQVQAGFN----------------------  16
AMSG11662     390 ITSGNIDGYVGKRVGNLTKVYSEELQ---------------------- 415

cons          817                                                    864

Q54BM7        433 --------STRKTL---KP----------------------------- 440
C300023       332 --------STRKTL---KP----------------------------- 339
P0A7B1         67 --QGSN-----------SH-----------------------------  72
A2VBB6        191 SKATAAL-RLRRQI---RP----------------------------- 205
Q2MEV6        304 --NLINV-QSRMAW---TP----------------------------- 316
A9U2N0        233 --NLIKL-QPRMAW---TP----------------------------- 245
Q01H21        119 --NLVKA-RGREVW---TP----------------------------- 131
A4RQI1         82 --NLVKQ-RGREVW---TP-----------------------------  94
A2SQZ9         89 --------EFSEVI---GS-----------------------------  96
D3B5H9        581 --------STRKSL---KP----------------------------- 588
E9CFK0        135 KASRAAMPERTTAATPATPSSQRPSDSRDSTQTSMDATSDWAVGALPS 182
EGG21828.1    570 --------STRKSL---KP----------------------------- 577
CMM026C       432 ------------------------------------------------ 431
F4PF87         17 -----KP-ENKAGL---TP-----------------------------  26
AMSG11662     416 ----MAA-DMGEGY---R------------------------------ 425

cons          865                                                    912

Q54BM7        441 --------------------------------------EEHYINMVLN 450
C300023       340 --------------------------------------EERYINLVLN 349
P0A7B1         73 --------------------------------------SRHLLGKIQS  82
A2VBB6        206 --------------------------------------RSQFEADLAD 215
Q2MEV6        317 --------------------------------------QQQL-KHVAK 325
A9U2N0        246 --------------------------------------QQQL-KHIAR 254
Q01H21        132 --------------------------------------NETL-RNVAV 140
```

```
A4RQI1        95 --------------------------------------QETL-QNVAA  103
A2SQZ9        97 --------------------------------------STQL-EMIYS  105
D3B5H9       589 --------------------------------------EERYINMVLN  598
E9CFK0       183 ELLPVALLHQARREQQAASQPDSGSSSTEAGNGQPDTNNSQL-HRLQS  229
EGG21828.1   578 --------------------------------------EEKYINMVLN  587
CMM026C      432 -----------------------------------------Y-ERISA  437
F4PF87        27 --------------------------------------KQQL-NAIAK   35
AMSG11662    426 ---------------------------------------PQL-VSVYE  433

cons         913                                               :     960

Q54BM7       451 TT-------------------------------------------RNL  455
C300023      350 TT-------------------------------------------RNL  354
P0A7B1        83 RV-------------------------------------------L-K   86
A2VBB6       216 AV-------------------------------------------RSI  220
Q2MEV6       326 EV-------------------------------------------RLM  330
A9U2N0       255 DV-------------------------------------------RLM  259
Q01H21       141 EV-------------------------------------------ERQ  145
A4RQI1       104 EV-------------------------------------------ERQ  108
A2SQZ9       106 RV-------------------------------------------IVL  110
D3B5H9       599 TT-------------------------------------------RNL  603
E9CFK0       230 SLERYAAAAAPDTSDSVAPTTSSVLQSNDKCMVLLPEYLQVNQHISQL  277
EGG21828.1   588 TT-------------------------------------------RNL  592
CMM026C      438 AI-------------------------------------------RDI  442
F4PF87        36 LN-------------------------------------------HTL   40
AMSG11662    434 TI-------------------------------------------HSM  438

cons         961                                                     1008

Q54BM7       456 INEIYNIYMNILAPELSNNNVFIIKY-SDLTEPEKIQLRGFFLQHVFP  502
C300023      355 INEMYNLYCNILQPELANNNVFIIKY-NELTEPEKVQLRAFFLQHVFP  401
P0A7B1        87 ADQEFDGLYNELLLEMARNQIFLINE-RQLSVNQQNWLRHYFKQYLRQ  133
A2VBB6       221 VNVQHEVLTETLLPALRGHGIELIPY-EALTPAQGASMTAYFRRSLFP  267
Q2MEV6       331 VDTQIACLTEEVLPALKKNGFNLVDY-EELHLHEKDQLRLYFKSSLEP  377
A9U2N0       260 VDTQIACLTEELLPALNKKEIKLVDY-DGLHLHEKDQLRLYFKSALEP  306
Q01H21       146 VDVQAALLEDHIVPALGEHGVRLEQY-DDLEEEVKQKLESYFEAQIEP  192
A4RQI1       109 VDAQAALLEDHIVPALKAHNIYLEQY-DDLSKDLRTKLGDYFEAQIEP  155
A2SQZ9       111 MRHMAQVWNSDLLPKLAAEGIFFIRY-SECTDEEKKAFKQEFEAEEFT  157
D3B5H9       604 INDKYNLFNNILLPELAANNVFLLSY-KDLQDSEKFQLRAFFLQHVFP  650
E9CFK0       278 ECRMRQLFARQLQPDLAAVGIDIVTNPNTLSVVERSEVIAQCETKLLP  325
EGG21828.1   593 INDKYNLFNNKIVPELAANNVNLLKY-DDLQDGEKYQLRAFFLQHVFP  639
CMM026C      443 TAQQESCFTDVILPALARYDMRILRY-ADLSMTQRAQMNAFFHARLFP  489
F4PF87        41 VDKQYEGFN-ALQQALADEEISILEM-DDLTIEERDTLEEYFDEQIFP   86
AMSG11662    439 TDEMGEVFS-SVTTQLTAHGVHVLRM-AELTPEELEPLREVFV-----  479

cons        1009           :   :    . .            .                 1056

Q54BM7       503 LMTPLVVDAGHPF-PN-------------------------------L  518
C300023      402 LMTPLVVDAGHPF-PN-------------------------------L  417
P0A7B1       134 HITPILINPDTDLVQF-------------------------------L  150
A2VBB6       268 LLTPMSVDQTHPF-PL-------------------------------L  283
Q2MEV6       378 ILTPLAVDPGHPF-PY-------------------------------I  393
A9U2N0       307 ILTPLAVDPGHPF-PY-------------------------------I  322
Q01H21       193 VLDPRAIDPCHPF-PF-------------------------------L  208
A4RQI1       156 VLDPRAIDPCHPF-PF-------------------------------L  171
A2SQZ9       158 ILRGDRFSDIHHD-EY-------------------------------L  173
D3B5H9       651 LMTPLVVDAGHPF-PN-------------------------------L  666
E9CFK0       326 HLRI-STKLPHPA-SLDAAVGTIHLFVGSAAAVSLAGQAESSSTTPFF  371
EGG21828.1   640 LMTPLVVDAGHPF-PN-------------------------------L  655
CMM026C      490 LLTPLTLDPTHPF-PL-------------------------------L  505
F4PF87        87 ILTPMAIDAYHPF-PM-------------------------------L  102
AMSG11662    480 ------------------------------------------------  479

cons        1057                                                     1104
```

```
Q54BM7         519 SNLSLNIAVLLKHDE---------------------------------  533
C300023        418 SNLSLNIAALLKHDE---------------------------------  432
P0A7B1         151 KDDYTYLAVEIIR-G---------------------------------  164
A2VBB6         284 QSHGIYLLVVLLNPE---------------------------------  298
Q2MEV6         394 GNLTSSIAVVLRDPY---------------------------------  408
A9U2N0         323 GNLTLSIAVVLRDPY---------------------------------  337
Q01H21         209 GSYSLSIAVELEDAF---------------------------------  223
A4RQI1         172 GSLSLSIAVELEDAF---------------------------------  186
A2SQZ9         174 RGFAMLV-----QTN---------------------------------  183
D3B5H9         667 SNLSLNVGVILQHDE---------------------------------  681
E9CFK0         372 PSWFRPSGAQDQAD----------------------------------  385
EGG21828.1     656 SNLSLNIAVVLQHDE---------------------------------  670
CMM026C        506 RSHSLYLAVLLKGAQ--LMPPSEHDAHDGDESPIPALSPPNASLDRSI  551
F4PF87         103 LNKSINLAVVLEDTYEVE------------------------------  120
AMSG11662      480 ------------YDG---------------------------------  482

cons          1105                                                  1152

Q54BM7         534 -----DTTRFVRIKVPQ-RIPRFVHIKQ-----RSNYSIIPMEEIILA  570
C300023        433 -----DTTRFVRIKVPQ-RIPRFVHIKQ-----RSPYSIIPMEEIILA  469
P0A7B1         165 -----DTIRYALLEIPSDKVPRFVNLPPE--APRRRKPMILLDNILRY  205
A2VBB6         299 ----SGVTRRVYFRVPA-VKPRLLPVT--------PLQFLPVEQLVVA  333
Q2MEV6         409 ----DDAVQFAIVSVPS-GLERWKSLAFSKNDEYNMNTFVSLEDIIIN  451
A9U2N0         338 ----DDAVQFAIVSVPS-GLERWKSLEFTETDEYNRNAFVPVEDIIIN  380
Q01H21         224 -----NQDKFAIVSVPA-ALDRWVRVPSP--KGESTQRFLPLEQLIEA  263
A4RQI1         187 -----KQEKFAIVSVPA-ALERWIRVGDS--KDSSEQRFLPLEQLIEA  226
A2SQZ9         184 ----KGRAVIPVQRIID-ERGRIVPVGV-----R-KNTFIFREDILRK  220
D3B5H9         682 ----EGHTRFVRIKVPL-KIPRFVHIKQ-----RSNYSLIPMEEIILA  719
E9CFK0         386 ----EQQQQFLWIEIPP-VAGKWLYLARK----NDRHAFVPVEVAVAL  424
EGG21828.1     671 ----EGHTRFVRIKVPL-KIPRFVHIKQ-----RQPYSLIPMEEIILA  708
CMM026C        552 ATALPSDTHVAWVRVPS-SCSRFITVD-------NEKRFLPVEEIIIQ  591
F4PF87         121 ----FKSRKNAIVQVPA-LLDRYIQIGD-----T--NRYILLEDIISH  156
AMSG11662      483 ----KGSLRFVYLPVPL-DLGRWIQISSP----RGRFCYVRSEDLIRH  521

cons          1153               :      :   :             :  :  :    1200

Q54BM7         571 NLD----------TLFPN---TKI---------------LTKSLFRVS  590
C300023        470 NLD----------TIFPN---TEI---------------VSKSLFRVT  489
P0A7B1         206 CLD----------DIFKGFFDYDA---------------LNAYSMKMT  228
A2VBB6         334 HLP----------LVCEG---MTI---------------LRWYAFRVT  353
Q2MEV6         452 NLD----------LLFGG---MEI---------------MSAHVFRTT  471
A9U2N0         381 NLD----------LLFGG---MEI---------------KGAYAFRTT  400
Q01H21         264 NID----------KLFRG---CSI---------------RATHVFRVT  283
A4RQI1         227 NME----------KLFRG---CNI---------------RDTHVFRVT  246
A2SQZ9         221 HIP----------SLFPD---ETV---------------YAVMTVRLT  240
D3B5H9         720 NLD----------TLFPN---TKI---------------LTKSLFRVT  739
E9CFK0         425 SLRQVAVEHPQLLSLFAK---LAILPQSTGTSLSPPYICAGVHILRDS  469
EGG21828.1     709 NLD----------TLFPN---TKL---------------LTKCLFRVT  728
CMM026C        592 NLD----------ALFEN---VRV---------------IAAYPFRVT  611
F4PF87         157 FLY----------KFFKG---YRV---------------HSVTEFRIT  176
AMSG11662      522 FLP----------TMYPL---HKI---------------VAAHVFR--  539

cons          1201  :            .                             .:   1248

Q54BM7         591 -RHNDLKLSG--EDQAND---LLELIKTELHKRKFAPMVRLEVS----  628
C300023        490 -RHNDLKLNN--EDQAND---LLELIKTELHKRRFAPMVRLEVS----  527
P0A7B1         229 -RDAEYDLV---HEMEAS---LMELMSSSLKQRLTAEPVRFVYQ----  265
A2VBB6         354 -RNTKLVVDDTLFGESDN---LLDYVWEEVHRRRSAPATRLEVT----  393
Q2MEV6         472 -RNADVARN---EEEAED---LLEMITDEMRELRFAPFVRLEVD----  508
A9U2N0         401 -RNADVARN---EEEAED---LLEMIADEMRELRFAPFVRLEVD----  437
Q01H21         284 -RNADIERN---EDQAED---LLEMIADEVRERRFASFVRLEVQ----  320
A4RQI1         247 -RNADIERN---EDEAED---LLEMMADEVRERRFASFVRLEVQ----  283
A2SQZ9         241 -RDSDLDLKG---DDADD---LISAIIDAPKTLAKRLPSRLETL----  277
D3B5H9         740 -RHYDLKLN---EDEAND---LLELIKTELHKRKFAPMVRLEVS----  776
```

```
E9CFK0         470 HPHADTPRQD---DDSASEIPIPESMLQLLQERQDARIVRFVAQPLCT  514
EGG21828.1     729 -RHYDLKLN---EDDAND---LLELIKTELHKRKFAPMVRLEVS----  765
CMM026C        612 -RNTKLELDKIELNESED---FLKIVEENLYSRQRRAAVRLEVS----  651
F4PF87         177 -RNADMTIH---EEGARD---LLKEIEKELRKRKWGAAVRLEVR----  213
AMSG11662      540 -----------------D---VLELMSSGIDMRRYAQVVRLEVT----  563

cons          1249                  .   . . :             *:       1296


Q54BM7         629 ------------------------------HNMPAE-ILDMLK-----  640
C300023        528 ------------------------------QNMPPE-ILDMLK-----  539
P0A7B1         266 ------------------------------RDMPNA-LVEVLR-----  277
A2VBB6         394 ------------------------------STMPMA-DVARLA-----  405
Q2MEV6         509 ------------------------------CKMPLE-VVQRLT-----  520
A9U2N0         438 ------------------------------REMPLE-VVQRLV-----  449
Q01H21         321 ------------------------------DTMPEQ-VRDELI-----  332
A4RQI1         284 ------------------------------DTMPEY-IRDKLI-----  295
A2SQZ9         278 ------------------------------DTMPFG-YMAPLV-----  289
D3B5H9         777 ------------------------------STMPKN-IIEMLR-----  788
E9CFK0         515 CSLQSPCPACAPADTMRAVYQTWLWSLMAQLDLPLE-SVLLVLASGHR  561
EGG21828.1     766 ------------------------------ASMPKD-ILDMLI-----  777
CMM026C        652 ------------------------------RDMPDV-LTTLLR-----  663
F4PF87         214 ------------------------------KGKYDDTIIRFLL-----  226
AMSG11662      564 ------------------------------HDVHPD-VIEMLS-----  575

cons          1297                                          :       1344


Q54BM7         641 --------TQL-AL-DAYDVYVI--NGP--LGLQDLFEL---CKLN-L  670
C300023        540 --------TQL-AL-DEYDVYSI--NGP--LGITDLFEL---CKLN-L  569
P0A7B1         278 --------EKL-TI-SRYDSIVP--GGR-YHNFKDFINF---PNVG-K  308
A2VBB6         406 --------AEL-AL-DATDVYTL--PGP-LLGLADCMSI---AFAP-V  436
Q2MEV6         521 --------MEL-GLSDKDDVYAI--CGP--MALGELDSLV--GKCNLT  553
A9U2N0         450 --------MEL-GLNDLNDVYAV--CGP--IALGELASLP--VKLEVN  482
Q01H21         333 --------ESL-DGITLTDVITVPHRAP--LGFGTIDSIP--IDFGMD  367
A4RQI1         296 --------DSL-DGITLTDVITVPHRAP--LGFGTIDSLP--VNFGMD  330
A2SQZ9         290 --------DAL-SL-VPELVYDM--EAP--LGLADLKDF----PVK-R  318
D3B5H9         789 --------IQL-RL-DEADVYII--NGP--LGLADLFEL---CKLN-L  818
E9CFK0         562 AASAHTATSALAGL-PELRIVEQ--SAS-LLHLHRLDQLV--SAVS-N  602
EGG21828.1     778 --------TQL-HL-DDADVYII--DGL--LGLADLFEI---CKLN-L  807
CMM026C        664 --------EQL-NL-EPLAMYRS--RAP-IIGLNDCFML---TNVN-I  694
F4PF87         227 --------NVL-EI-HKDDLYIV--DGP--LDLTFLFRFYKDAQVD-H  259
AMSG11662      576 --------FHL-NI-ERISIYSL--KFEELIGLADMMFF---VRLP-L  607

cons          1345           *                     :     :          1392


Q54BM7         671 PHLKFQPWVPHIPSRLVNL-----------AKYPSEDVFSVIRKGELL  707
C300023        570 PHLKFQPWVPHIPQKLVNL-----------ARYPSEDIFSVIRKGEFL  606
P0A7B1         309 ANLVNKPLPRLRHIW---F-----------DKAQFRNGFDAIRERDVL  342
A2VBB6         437 PHLQHPPHEPVTPPRFRGLEDKL--------QSDPGRIFSVIRKGDVL  476
Q2MEV6         554 SPLVYTLWTSKTHPRLQ-----------------GADVFEVIKKGDIL  584
A9U2N0         483 SSLVYTPWTPKTHPRIQ-----------------GADIFEVIRKGDIL  513
Q01H21         368 VAMLYSPWSPRTHSALD-----------------GKSIFEAISEGDIL  398
A4RQI1         331 IEMLYPPWSPRTHPRLEQI------------RGSEGTIFSAISEGDIL  366
A2SQZ9         319 PALKFPTYTASLPSGL------------------SGKIFSAIARRDRF  348
D3B5H9         819 PHLKFEPWVPHIPNRLANL-----------SKIPSQDIFSVIRKGELL  855
E9CFK0         603 PQLKFPPWRPVVHPLFAFLPNSL----NEMNPEIADRFFERLTERDVM  646
EGG21828.1     808 PHLKFEPWIPHIPSRLVNL-----------SKYPQQDIFSVIRKGELL  844
CMM026C        695 PALRYPPWRFKTHSILAGYKY-------GVQSGHTAHMFELIRHNDVL  735
F4PF87         260 ENLIYQTLIPQRPQDID----------------SKESIFEAAEKRDIF  291
AMSG11662      608 PALKYRPWRPIPHPSFRSLTQLTNGRPRPLTPDIAASVFDLVSEQDIY  655

cons          1393   :                                   *.   . :   1440


Q54BM7         708 VNLPYLSFNSSVQFFIESAVKDPKVLAIKIAIYRTNSNSQLIRALCEA  755
C300023        607 VNLPYLSFNSSVQFFIESAVKDPKVLAIKIAIYRTNSNSQLIRALCEA  654
```

```
P0A7B1       343  LYYPYHTFEHVL-ELLRQASFDPSVLAIKINIYRVAKDSRIIDSMIHA  389
A2VBB6       477  VQYPAHSFTGSALLFLRAAARDPRVRVIKQVLYRGGSHSPLVASLIRA  524
Q2MEV6       585  LHHPYHSFVTSTQHFVEAAANDPKVAAIKATLYRTNNDSPVISALAKA  632
A9U2N0       514  LHHPYHSFVTSIQHFVEAAANDPKVMAIKATLYRTSNDSPVISALAKA  561
Q01H21       399  VHHPYTSFATSTQAFIEEAARDPDVLSIKSTLYRTSDNSPIVRALIKA  446
A4RQI1       367  VHHPYVSFATSTQAFIEEAARDPDVLSIKSTLYRTSDNSPIVRALIKA  414
A2SQZ9       349  MFTPYNSFDGLI-RFMNAAAEDPTVIKIQMTLYRLGSESPVIDALLAA  395
D3B5H9       856  INLPYVSFNSSVQFFIESAVKDPKVLAIKIAIYRTSSNSQLIRSLCEA  903
E9CFK0       647  AHHPFHSWTHSVLAMLRAACLDPKVTDITISLYRVAENSAVVQSLVEA  694
EGG21828.1   845  VQLPYVSFNSSVQLFIESAVKDPKVLAIKIAIYRTSSNSQLIRALCEA  892
CMM026C      736  FSFPIHSFDETTLRFLESAVRDPAVRLMKMVLYRCGNNSPVVKLLMEA  783
F4PF87       292  LHHPYESFGPVVD-LITEAAIDPEVMAIKQTLYRVSGHSPIIKGLKQA  338
AMSG11662    656  IQFPYVSFESTVQIFVQSAAIDPQVLSIKMTLYRLASQDAVVDALINA  703

cons        1441     *  ::      ::   *  ** *  :   :**   .. ::  :  *  1488

Q54BM7       756  AS--HKEVMVLVDLKASGDEEQNTKFARLLEQAG-CHVSYGLVGLKTH  800
C300023      655  AS--HKEVMALIDLKASGDEEQNTKFARLLEQAG-CHVSYGLVGLKTH  699
P0A7B1       390  AHN-GKKVTVVVELQARFDEEANIHWAKRLTEAG-VHVIFSAPGLKIH  435
A2VBB6       525  AKS-GKEVTVLVELKASFDEVQNSEYARRLQRAG-CNVSYGLVGLKTH  570
Q2MEV6       633  AEA-GKQVAVLVELKARFDEVRNMGFAQKLEDAG-CNVAYGLVGLKTH  678
A9U2N0       562  AES-GKQVAVLVELKARFDEERNIGFAQKLEDAC-CNVVYGLIGLKTH  607
Q01H21       447  AEN-GKQVAVLVELKARFDEARNVGFAERLETAG-CNVAYGVKGVKTH  492
A4RQI1       415  AEN-GKQVAVLVELKARFDEARNVGFAQRLETAG-CNVAYGVKGVKTH  460
A2SQZ9       396  AKN-KKDVTAVVELKASFDEETNFRWATKLKEGG-VNVIMGYPGVKVH  441
D3B5H9       904  AS--HKEVSVLVDLKASGDEEQNTKYARLLEQAG-CHVSYGLVGLKTH  948
E9CFK0       695  ATTFQKRVTCIVELRASLDEERNAWAARRLRDTGRCRVLFGRQAWIMH  742
EGG21828.1   893  AS--HKEVSVLVDLKASGDEEQNTKYARLLEQAG-CHVSYGLVGLKTH  937
CMM026C      784  ARR-GIDVNVLVELKASFDEDQNVLYARMLQEAG-CNVAYGVKGYKTH  829
F4PF87       339  AEN-GKQVTVLVELKARFDEEQNVHWAKELEKAG-CHVIYGMNFLKTH  384
AMSG11662    704  VAN-GKEVTVIVELKASYDEARNLAYATALEQAG-ATVCYGLQGMKTH  749

cons        1489  .      *  :::*:*  **  *   *  *       *  .      *  1536

Q54BM7       801  AKIAMVVREEE-NG--------LREYLNISTGNYNASTSDVYADICLF  839
C300023      700  SKIAMVVREEE-NG--------LREYLHISTGNYNASTADVYADIGLF  738
P0A7B1       436  AKLFLISRKEN-GE--------VVRYAHIGTGNFNEKTARLYTDYSLL  474
A2VBB6       571  CKTMVVVREEEAGG--------LRAYVNVATGNFNPTTAKLYTDMALF  610
Q2MEV6       679  CKCIMVVRKED-DG--------LRTYVHIGTGNYNPRTASVYTDFGLL  717
A9U2N0       608  CKCILVVRKED-DG--------LRTYVHIGTGNYNPRTASVYTDFGLL  646
Q01H21       493  SKASLVVRREG-KK--------LVKYVHIGTGNYNPSTAGIYTDFGLL  531
A4RQI1       461  SKASLVVRREG-KK--------LVKYVHIGTGNYNPSTAGIYTDFGLL  499
A2SQZ9       442  AKCCLVTRIEN-GQ--------IVRYANISTGNYNAKTAKIYSDISIF  480
D3B5H9       949  AKIAMVVREEE-NG--------LRMYNHYSTGNYNASTADIYADMGLF  987
E9CFK0       743  AKLLVVTRREG-GDRPGTAAPTERLYAQIGSGNYNESTCDRYTDLSYF  789
EGG21828.1   938  CKIAMVVREEE-NG--------LREYLHYSTGNYNSSTADIYVDMCLF  976
CMM026C      830  AKLILVVREEN-HA--------LVSYCNVATGNYNATTAKIYTDFSLF  868
F4PF87       385  SKITLIVRKRK-EK--------IERFVHLGTGNYNDSTADLYTDMGLV  423
AMSG11662    750  GKIIRVVRSQP-DG--------LRTYSLVATGNFNSTTAPLYIDMGLL  788

cons        1537   *   : * .               :   .:**:*  *.  * *   .  1584

Q54BM7       840  SCDPDLGEDMCNLFNYLTG--------------------------YSR  861
C300023      739  SCDPELGEDMCNLFNYLTG--------------------------YSR  760
P0A7B1       475  TADARITNEVRRVFNFIEN--------------------------PYR  496
A2VBB6       611  TCAPAIAADVLDLFNALTG--------------------------YSR  632
Q2MEV6       718  SCDPDLGMDVADLFKYLTG--------------------------YHR  739
A9U2N0       647  SCDPDLGMDVINLFKYLTG--------------------------YHR  668
Q01H21       532  SRDDELGDDVSNLFKFLMG--------------------------HHY  553
A4RQI1       500  SRDDQLGDDVSNLFKFLMG--------------------------HHY  521
A2SQZ9       481  TADEDICTDAAALFSMFSG--------------------------SVP  502
D3B5H9       988  SCDQQLGADMCDLFNYLTG--------------------------YSR 1009
E9CFK0       790  TSNPLITAEITQLLARVAAEGVPIWQEVVLWLSALLAALVLYVAAIFS  837
EGG21828.1   977  SCDQQLGADMCDLFNYLTG--------------------------YSR  998
CMM026C      869  TSREDICADVTDLFNVFTG--------------------------YSA  890
```

```
F4PF87         424 TTKRKFGIDATNFFNYLSG--------------------------FTE  445
AMSG11662      789 TAHPEIGHELGQVFNYLTS--------------------------YCG  810

cons          1585 :    :  :   .:  .                                1632


Q54BM7         862 VS------------------------------SFKKLLIAPMNMRSTL  879
C300023        761 IT------------------------------SFKKLVIAPMNMRSTL  778
P0A7B1         497 PV------------------------------TFDYLMVSPQNSRRLL  514
A2VBB6         633 KR------------------------------NYRRLLVAPVSMLDRV  650
Q2MEV6         740 QV------------------------------AYRKLLVAPGTMRNEF  757
A9U2N0         669 QV------------------------------AYLKLLVSPGTMRNEF  686
Q01H21         554 QE------------------------------RFKKLLVAPVRMQKEF  571
A4RQI1         522 QE------------------------------RFNKLIVAPLRMQNEF  539
A2SQZ9         503 AP------------------------------AYRRLIVSPHSMNAQL  520
D3B5H9        1010 IT------------------------------SFRKLLIAPMNMRSTL 1027
E9CFK0         838 LSFFKQLLASKSETPTEPVPTSALPASEEAEFPTQHVLVSPFNMRDRF  885
EGG21828.1     999 IT------------------------------SFRKLLIAPMNMRSTL 1016
CMM026C        891 KR------------------------------SFRKLLVSPVNMRDRF  908
F4PF87         446 KP------------------------------DFNHLSMAPFDIRADF  463
AMSG11662      811 RG------------------------------KFKKLLVSPFNTTATF  828

cons          1633                                     : ::*      . 1680


Q54BM7         880 IQLIDNEAKNAREGKD--------ATINAVMNGLDDKRLVNALYQASI  919
C300023        779 IQLIDNEAKNAREGKE--------ATINAVMNGLDDKRIVNALYQASI  818
P0A7B1         515 YEMVDREIANAQQGLP--------SGITLKLNNLVDKGLVDRLYAASS  554
A2VBB6         651 VAMIAAEAAHARAGRP--------ARIIAQINGLTEPEIIRHLYAASQ  690
Q2MEV6         758 IWLIEREIANAQAGKP--------ASIIVKCNGLDDQVMVTKLYEASK  797
A9U2N0         687 VWLIEREIVNAQMGKP--------ASIIVKCNGLDDQVMVTKLYEASK  726
Q01H21         572 VDMIEREAENALRGKP--------ASIIAKMNGLDDPTICAALYRASQ  611
A4RQI1         540 VEMIEREAENALKGKP--------ASIIAKMNGLDDAVICAALYRASQ  579
A2SQZ9         521 IAKIMREAEV--SGKQ--------GRIIMKMNTLTDREIINALYAASE  558
D3B5H9        1028 IQLIETEAQNAREGKE--------ASILAVMNGLDDKKIVNALYHASV 1067
E9CFK0         886 LALIAHETEMAKSKAKSDSSDDGLPDIFACCNALDDGAIVEALIQAAQ  933
EGG21828.1    1017 IQLIETEARNAREGKE--------ASILAVMNGLDDKRIVNALYHASV 1056
CMM026C        909 LELIQREIDNARAGYP--------ARIICQCNGITEVLITQRLYEASM  948
F4PF87         464 IRYVNEEINFHQLRGN--------GHIIAKMNSLTDKEIIRKFYEASQ  503
AMSG11662      829 VKLIKKETRNAEAGKK--------ALIIARLNGLTDKTITAALYEAAA  868

cons          1681    :  *                   *    * : :  :   :  *:  1728


Q54BM7         920 AGVKITLVVRGRCRILPGIKGISENIKVISILGRFLEHSRIYCFHNNG  967
C300023        819 AGVKITLVVRGRCRILPGIKQISENIRVISVLGRFLEHSRIYCFHNNG  866
P0A7B1         555 SGVPVNLLVRGMCSLIPNLEGISDNIRAISIVDRYLEHDRVYIFENGG  602
A2VBB6         691 AGVIIDLIIRGPCRLRPGLPGRSENIRVFSWVGRFLQHRRVFYFSAGG  738
Q2MEV6         798 AGVNIDCIVRGMCRIRTGIPNVSDNIRVVSILGRFLEHHRIFRFENGG  845
A9U2N0         727 AGVKVDCIVRGMCRIRTGIPNVSDNIRVVSIIGRFLEHHRVMRFENGG  774
Q01H21         612 AGVKINLIVRGICRVRPGIPGVSENIRVVSIIGRFLEHHRAFRFENNG  659
A4RQI1         580 AGVKIHLIIRGICRVRPGIPGVSENIRVVSIVGRFLEHHRVFRFENNG  627
A2SQZ9         559 MGVQIDLAVRGVCMLRPGVPGLSETIRVISVVGRFLEHARFYYFENSG  606
D3B5H9        1068 AGVTIKLVVRGRCRILPGIPGISHNIQVISILGRFLEHSRIYYFCNGG 1115
E9CFK0         934 AGANIFLSVRGPSRITPKMRNIR-SLRIISVVGRFLEHTRFFYFRNGW  980
EGG21828.1    1057 AGVTIKLVVRGRCRILPGIPGISHNISVISILGRFLEHSRIYYFSNGG 1104
CMM026C        949 AGVRVDLIVRGICRVRPGITGISENIRVVSILGRFLLHARVFYFANAR  996
F4PF87         504 AGVKIDLIVRGICCLKPGIEGVSENIRVRSIVGRFLEHSRIYYFHSDG  551
AMSG11662      869 AGVKIELLVRGMARIRPGIPGKTENVRVMSYVGRFLEHSRIYYFYNDG  916

cons          1729  *. :   :** . : . :     .:   * :.*:* * *   *    1776


Q54BM7         968 --------------------------KPKAYIASADWLHRNLKRRVEV  989
C300023        867 --------------------------KPKAYIASADWLHRNLKRRVEV  888
P0A7B1         603 --------------------------DKKVYLSSADWMTRNIDYRIEV  624
A2VBB6         739 --------------------------SDLYFISSADWRSRNLNDRIEV  760
Q2MEV6         846 --------------------------DAEYYMGSADWMTRNLTRRVEV  867
```

```
A9U2N0        775  --------------------------KAEYYMGSADWMNRNLTRRVEV  796
Q01H21        660  --------------------------EPEFYMGSADMMRRNLLRRVEV  681
A4RQI1        628  --------------------------SPEFYMGSADLMRRNLLRRVEV  649
A2SQZ9        607  --------------------------DPELYIGSPDMMPRNLHRRIEI  628
D3B5H9       1116  --------------------------TPKAFIASADWLHRNLKRRVET 1137
E9CFK0        981  SGWKDANGEPSSNPNSNDSQLALPRSGPMYIVGSADWAHSKLSKRIEI 1028
EGG21828.1   1105  --------------------------APKAYIASADWLHRNLKRRVET 1126
CMM026C       997  E------------------------AKPEYYIGSADWRTRNLLARLEA 1020
F4PF87        552  --------------------------DEKIFLSSADLMTRNMAKRVEL  573
AMSG11662     917  --------------------------KPRYYLGSADWMTRNLKFRIEV  938

cons         1777                                 :.*.*    ::  *:*  1824
                                                    ↑ ↑          ↑

Q54BM7        990  MVPVDDANNIKQL-YEIINVYCN-DS-NAWEMLSD--GRYKKRSSPID 1032
C300023       889  LVPVEDPNNIKQL-YDIIKVYVN-DT-NAWDMLPD--GRYKKRSVPID  931
P0A7B1        625  ATPLLDPRLKQRV-LDIIDILFS-DTVKARYIDKELSNRYVPRGNRRK  670
A2VBB6        761  ATPVDDPRIKRKL-RKSLNFFLN-DRLHTWQLASD--GRYYMPRAPLT  804
Q2MEV6        868  VTPVEDEALQKEL-QEMLDACLC-DRRKAWEMRPD--GRYQLLGVGNM  911
A9U2N0        797  VVPVEAEPLQKEL-QEMLDACLC-DRRNAWEMRPD--GRYQLLGIGTM  840
Q01H21        682  ITRVLDPRIQAEL-QEVLDACLA-DQVLAWEMGSD--GRYYKTEASRE  725
A4RQI1        650  ITRVYDPKIQTEL-QEVLDACLT-DQVLAWEMGSD--GRYYRTPTSRQ  693
A2SQZ9        629  LCPILDKRIKKKVFIQKLIPEFMY-DSVKGHTLDAY--GRYHPPERKPG  673
D3B5H9       1138  LVPVEDPNNIKEL-RHVIDVYCN-DP-NAWFMLPD--GRYKKTSSSDF 1180
E9CFK0       1029  ALPVRDPLIASHL-ERTMRLYLS-TS-TACQMQED--GSYSVQSATTA 1071
EGG21828.1   1127  LVPVEDPANIKEL-RSIIDVYIN-DP-TAWEMLPD--GRYKKVASNEH 1169
CMM026C      1021  VAPVEDKYLQQTL-WRHLNRLLS-DSVNGWEMLPD--GRYVKGESEAL 1064
F4PF87        574  MFPIYEAPVKKRV-IEVLLITLQ-DNIKAREQASD--GTYHYVEAEPG  617
AMSG11662     939  LTPVYDKNIQACI-MSELQLYLSPERANVWDMQPD--GRYQRRQPPRT  983

cons         1825     :        .    :                   . *          1872

Q54BM7       1033  ----------------------------EDSQ---------------- 1036
C300023       932  ----------------------------EDSQ----------------  935
P0A7B1        671  V----------------------------RAQ----------------  674
A2VBB6        805  TV-G----------------PPAVDEASSATA----------------  819
Q2MEV6        912  KD-P----------------P--RKHIPQVAS----------------  924
A9U2N0        841  KT-P----------------P--REHTPQAAS----------------  853
Q01H21        726  V--A----------------A--NHLASDPGK----------------  737
A4RQI1        694  V--A----------------A--NHLAHDPGK----------------  705
A2SQZ9        674  N---------------------------LSS-----------------  677
D3B5H9       1181  AA--------------------------LGAQ---------------- 1186
E9CFK0       1072  R---------------------------ADDV---------------- 1076
EGG21828.1   1170  AS--------------------------LASQ---------------- 1175
CMM026C      1065  GHLRDAIMAARREAEAEALKPPAQAPVSADEVTPGSSEAEHLPVVRKA 1112
F4PF87        618  EA-L------------------------IDSQ----------------  624
AMSG11662     984  TT-N----------------RMGVWGIMMDSV----------------  998

cons         1873                                                    1920

Q54BM7       1037  ------------------------------------------------ 1036
C300023       936  ------------------------------------------------  935
P0A7B1        675  ------------------------------------------------  674
A2VBB6        820  -------------------------------ATLAGFARPPLRFEQPP  836
Q2MEV6        925  -------------------------------PKL--------------  927
A9U2N0        854  -------------------------------SKL--------------  856
Q01H21        738  -------------------------------GKL--------------  740
A4RQI1        706  -------------------------------GRL--------------  708
A2SQZ9        678  ------------------------------------------------  677
D3B5H9       1187  ------------------------------------------------ 1186
E9CFK0       1077  ------------------------------------------------ 1076
EGG21828.1   1176  ------------------------------------------------ 1175
CMM026C      1113  GCVPVRLNRRHNTRRDDIGTRYEVLLITSTSSS--FIARHPND----- 1153
F4PF87        625  ------------------------------------------------  624
AMSG11662     999  -------------------------------EVL-------------- 1001
```

```
cons          1921                                                   1968

Q54BM7        1037 ------------------------------------------------ 1036
C300023        936 ------------------------------------------------  935
P0A7B1         675 ------------------------------------------------  674
A2VBB6         837 PSL------GGGSSYAPPVDAFAEFDEGEEDDDDDYDE-LA-----AV  872
Q2MEV6         928 ---------------------------------------IAAVESMGL  936
A9U2N0         857 ---------------------------------------AATVENKGL  865
Q01H21         741 ---------------------------------------MKTSAEVGL  749
A4RQI1         709 ---------------------------------------MKMAATDGL  717
A2SQZ9         678 ------------------------------------------------  677
D3B5H9        1187 ------------------------------------------------ 1186
E9CFK0        1077 ------------------------------------------------ 1076
EGG21828.1    1176 ------------------------------------------------ 1175
CMM026C       1154 --SRVSEIPDGGITWVFPKGSMAY------GEDGRSAALREALEEAGV 1193
F4PF87         625 ------------------------------------------------  624
AMSG11662     1002 ---------------------------------------DA-----G- 1004

cons          1969                                                   2016

Q54BM7        1037 -----------------TQFM--------------------------- 1040
C300023        936 -----------------SYFM---------------------------  939
P0A7B1         675 -----------------LAI---Y------------------------  678
A2VBB6         873 EGGIWRVPAAVPGRAAVADYVRNN----------------SAPE----  900
Q2MEV6         937 HEGLMEIVAKRCRKAK-KVNLRK-------------------------  958
A9U2N0         866 HEGLMEIVAKRCRKAR-KLIMGK-------------------------  887
Q01H21         750 HEALMDDTLKRMKRVDKRSDLVKLATKKVKQVTPR----A----PKET  789
A4RQI1         718 HVALMADTLKMMKRSDKRANLATLATKKVKKV-ER----A----PKEV  756
A2SQZ9         678 ----------------------QQ------------------------  679
D3B5H9        1187 -----------------NVFI--------------------------- 1190
E9CFK0        1077 ----------------------QV------------------------ 1078
EGG21828.1    1176 -----------------NVFV--------------------------- 1179
CMM026C       1194 SGELGPLLSVSTKRKRRTVVMTEFYLLHVKQQ-LSQWGESSQRHR--- 1237
F4PF87         625 -----------------MTL---Y------------------------  628
AMSG11662     1005 ---------------IQTSHLHKF------------------------ 1013

cons          2017                                                   2064

Q54BM7        1041 -------------------------------NQ--------------- 1042
C300023        940 -------------------------------RQ---------------  941
P0A7B1         679 -------------------------------D----------------  679
A2VBB6         901 -------------------------------RD---------------  902
Q2MEV6         959 --------------------QD------G-------------------  961
A9U2N0         888 --------------------RT------E-------------------  890
Q01H21         790 FARTLKTNSLEVN--QDLVVTD------DQSKATQEKRPNFDDMIH--  827
A4RQI1         757 RARVLKTNAPEVNDKTDVIEQQ------VQAKAPENTRPKFEDVIS--  796
A2SQZ9         680 ------------------------------------------------  679
D3B5H9        1191 -------------------------------KS--------------- 1192
E9CFK0        1079 ------------------------------------------------ 1078
EGG21828.1    1180 -------------------------------RH--------------- 1181
CMM026C       1238 -RWFTLD------EAANVITKEYLLEALMKARQALGETPTSQPTSPPA 1278
F4PF87         629 -------------------------------DM---------------  630
AMSG11662     1014 -------------------------------RS--------------- 1015

cons          2065                                                   2112

Q54BM7        1043 ------------TN-QKHPVIWSK 1053
C300023        942 ------------TN-QQHPVIWTK  952
P0A7B1         680 ---------------YIKSLEQPE  688
A2VBB6         903 ------------AF-AISGGDTSS  913
Q2MEV6         962 ------------TAMKPRRQHDSS  973
A9U2N0         891 ------------ASRKLVIKTTAV  902
Q01H21         828 -------ADQKENV--DSENKRDS  842
A4RQI1         797 -------VEEGVIMRSVEDSADES  813
```

```
A2SQZ9          680 -------------KF-I-AMQKVWR  689
D3B5H9         1193 -------------TY-QKHPVIWSK 1203
E9CFK0         1079 -------------SL-MKLVQQSRL 1089
EGG21828.1     1182 -------------TI-NTHPIIWSK 1192
CMM026C        1279 PPAPPPAAAAAAAFESTTNGGKKH 1302
F4PF87          631 ----------------AYDVLEDEE  639
AMSG11662      1016 -------------QR-KWDATPVAP 1026

cons           2113                           2136
```
