# Supplementary material for: Horizontal transfer of bacterial polyphosphate kinases to eukaryotes: implications for the ice age and land colonisation
Source: BMC Res Notes. 2013 Jun 5;6:221. doi: 10.1186/1756-0500-6-221 (PMC3680246; doi:10.1186/1756-0500-6-221)

Horizontal transfer of bacterial polyphosphate kinases to eukaryotes: implications for the ice age and land colonisation.

Additional File 2. Phylogenetic analysis of PPK1 including closest matching cyanobacteria. Numbers on the branches indicate bootstrapping values out of 100 calculated for maximum likelihood. Colours of eukaryotes indicate major taxonomic groupings (blue- non-photosynthetic eukaryotes, green- plants and green algae, red- red algae, purple- cyanobacteria).

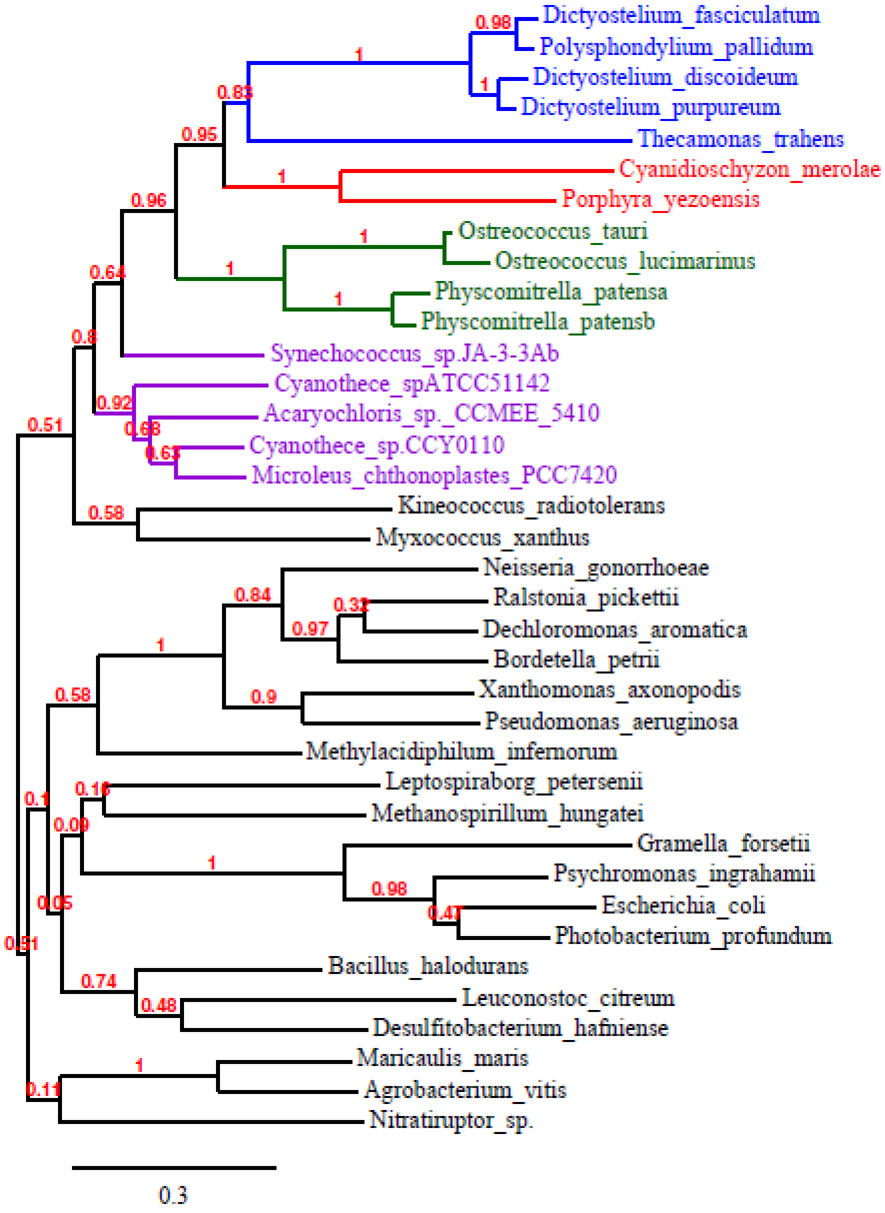

Supplement: Additional file 2 — Phylogenetic analysis of PPK1 including closest matching cyanobacteria. Numbers on the branches indicate bootstrapping values out of 100 calculated for maximum likelihood. Colours of eukaryotes indicate major taxonomic groupings (blue- non-photosynthetic eukaryotes, green- plants and green algae, red- red algae, purple-cyanobacteria). [file 1756-0500-6-221-S2.pdf]
