# Supplementary material for: Horizontal transfer of bacterial polyphosphate kinases to eukaryotes: implications for the ice age and land colonisation
Source: BMC Res Notes. 2013 Jun 5;6:221. doi: 10.1186/1756-0500-6-221 (PMC3680246; doi:10.1186/1756-0500-6-221)
Supplement: Additional file 4 — Representative species taken from bacterial taxa for phylogenetic analysis. [file 1756-0500-6-221-S4.doc]

**Additional File 4. Representative species taken from bacterial taxa for phylogenetic analysis.**

|  |  | PPK1 representative | PPK2 representative |
| --- | --- | --- | --- |
| **Archaea** | **Euryarchaeota** | *Methanospirillum hungatei* | *Methanosarcina barkeri* |
| **Bacteria** | **Actinobacteria** | *Kineococcus radiotolerans* | *Micrococcus luteus* |
|  | **Bacteroidetes/Chlorobi** | *Gramella forsetii* | *Bacteroides fragilis* |
|  | **Cyanobacteria** | *Cyanothece sp. ATCC 51142* | *Acaryochloris marina* |
| **Firmicutes** | **Bacillales** | *Bacillus halodurans* | *Bacillus weihenstephanensis* |
|  | **Clostridia** | *Desulfitobacterium hafniense* | *Heliobacterium modesticaldum* |
|  | **Lactobacillales** | *Leuconostoc citreum* | *Lactobacillus rhamnosus* |
|  | **Others** | *Methylacidiphilum infernorum* | *Rhodopirellula baltica* |
| **alpha subdivision** | **Others** | *Maricaulis maris* | *Paracoccus denitrificans* |
| **alpha subdivision** | **Rhizobiaceae** | *Agrobacterium vitis* | *Sinorhizobium meliloti* |
| **beta subdivision** | **Bordetella** | *Bordetella petrii* | *Bordetella avium* |
| **beta subdivision** | **Burkholderiaceae** | *Ralstonia pickettii* | *Burkholderia phymatum* |
| **beta subdivision** | **Neisseriaceae** | *Neisseria gonorrhoeae* | *Laribacter hongkongensis* |
| **beta subdivision** | **Others** | *Dechloromonas aromatica* | *Thauera sp* |
| **delta subdivision** |  | *Myxococcus xanthus* | *Desulfotalea psychrophila* |
| **epsilon subdivision** |  | *Nitratiruptor sp. SB155-2* | *Wolinella succinogenes* |
| **gamma subdivision** | **Enterobacteriales** | *Escherichia coli* | *Serratia proteamaculans* |
| **gamma subdivision** | **Others** | *Psychromonas ingrahamii* | *Candidatus Vesicomyosocius* |
| **gamma subdivision** | **Pseudomonadaceae** | *Pseudomonas aeruginosa* | *Pseudomonas aeruginosa* |
| **gamma subdivision** | **Vibrionaceae** | *Photobacterium profundum* | *Vibrio harveyi* |
| **gamma subdivision** | **Xanthomonadaceae** | *Xanthomonas axonopodis* | *Xanthomonas oryzae* |
|  | **Spirochaetales** | *Leptospira borgpetersenii* | *Leptospira biflexa* |
